# preCICE v2: A sustainable and user-friendly coupling library

**DOI:** 10.12688/openreseurope.14445.2

**Published:** 2022-09-30

**Authors:** Gerasimos Chourdakis, Kyle Davis, Benjamin Rodenberg, Miriam Schulte, Frédéric Simonis, Benjamin Uekermann, Georg Abrams, Hans-Joachim Bungartz, Lucia Cheung Yau, Ishaan Desai, Konrad Eder, Richard Hertrich, Florian Lindner, Alexander Rusch, Dmytro Sashko, David Schneider, Amin Totounferoush, Dominik Volland, Peter Vollmer, Oguz Ziya Koseomur

**Affiliations:** 1 Scientific Computing in Computer Science, Department of Informatics, Technical University of Munich, Garching, 85748, Germany; 2 Simulation of Large Systems, Institute for Parallel and Distributed Systems, University of Stuttgart, Stuttgart, 70569, Germany; 3 Usability and Sustainability of Simulation Software, Institute for Parallel and Distributed Systems, University of Stuttgart, Stuttgart, 70569, Germany

**Keywords:** multiphysics, multiphysics coupling, co-simulation, fluid-structure interaction, conjugate heat transfer, computer simulation

## Abstract

preCICE is a free/open-source coupling library. It enables creating partitioned multi-physics simulations by gluing together separate software packages.

This paper summarizes the development efforts in preCICE of the past five years. During this time span, we have turned the software from a working prototype -- sophisticated numerical coupling methods and scalability on ten thousands of compute cores -- to a sustainable and user-friendly software project with a steadily-growing community. Today, we know through forum discussions, conferences, workshops, and publications of more than 100 research groups using preCICE. We cover the fundamentals of the software alongside a performance and accuracy analysis of different data mapping methods. Afterwards, we describe ready-to-use integration with widely-used external simulation software packages, tests, and continuous integration from unit to system level, and community building measures, drawing an overview of the current preCICE ecosystem.

## 1 Plain language summary

Computer models are a key component of almost every research in science and engineering. They back up and connect the two traditional research methods; theory and experiment. Often, however, individual computer models are not enough to understand and describe phenomena and processes in science and engineering. Instead, multiple computer models need to be combined. Combining these models (also referred to as ’coupling’) is what the software package *preCICE* does.

As of 2022, over 100 research groups from various fields use preCICE to couple their computer models. This includes, for example, research groups in aerospace engineering, biomedical engineering, and climate and environmental research. Thus, preCICE’s sustainability (Can I reproduce my research results using preCICE in five years?) and usability (How much do I need to learn before I can start doing my research with preCICE?) are critical factors.

How this sustainability and usability has drastically been improved during the past five years, is the topic of this paper. It summarizes the work done by the authors (the development team of preCICE), including for example automatic testing, documentation, accuracy analysis of the methods implemented in preCICE, and ready-to-use integration with commonly used computer models.

## 2 Introduction

Flexible, modular simulation environments are key to many important application fields such as aerospace engineering ^1^, biomedical engineering ^2^, climate and environmental research ^3^, and many others. The need to provide smart mathematical and software solutions to combine different aspects of such simulations in a modular way is an emerging challenge ^4^. With increasing complexity of the respective software environments, the usability and maintainability of the involved software components become a critical issue, which is addressed by a growing research software engineering community (see, e.g., a recent position paper of the German community ^5^).

We present the software package preCICE, which enables black-box coupling of separate solvers for different types of numerical models. It has originally been developed for modular, so-called partitioned, simulations of fluid-structure interactions, i.e., the combination of a flow solver with a structural mechanics solver via a common surface at which forces and displacements are exchanged. Over the past ten years, preCICE has developed into a far more general tool for partitioned simulations, which can handle different types of coupling (weak/strong, explicit/implicit, surface/volume) and any type of equations. Examples range from fluid-structure-acoustics interactions ^6^, over blood flow simulation in the human body ^7^, free-flow porous media coupling ^8^, conjugate heat transfer ^9^, muscle-tendon system simulations ^10^, flow-particle coupling ^11^, to coupling between subsurface flow and planning tools for geothermal energy infrastructure ^12^. The coupling is not restricted to a pair of solvers, but has been extended to enable multi-component coupling of arbitrarily many solvers ^13^.

preCICE offers comprehensive functionality far beyond simple data exchange: It provides (i) a variety of mapping methods for data transfer between non-matching meshes of different solvers, (ii) quasi-Newton acceleration methods for iterative implicit coupling, and (iii) bottleneck-free point-to-point communication between processes of parallel solvers. preCICE was originally designed with surface coupling in mind, but most features can and have been used for volume coupling as well. All coupling numerics and communication are implemented in a library approach and are fully parallelized. The library can be used via a high-level application programming interface (API) in a minimally-invasive way (from the perspective of the coupled solvers).

The first version of preCICE, as presented by Bernhard Gatzhammer in his dissertation ^14^, used a server process per coupled solver and was, thus, not very efficient for the coupling of parallel solvers. Later works ^15– 17^ transformed preCICE to a fully parallel library with point-to-point communication, which shows good scalability on ten thousands of compute cores. A first overview paper of preCICE was published in 2016 ^18^, summarizing basic functionality, the API, and the user-specific configuration as well as showing example applications and validation cases. Semantic versioning of preCICE was introduced in 2017.

In this paper, we summarize new developments from 2016 to 2021, i.e., from the first reference paper ^18^ to the release v2.2, which is part of the first preCICE distribution v2104.0 ^19^ – a complete ecosystem of preCICE components. We present an overview of the functionality of preCICE in Section 3, in particular the numerical coupling methods, i.e., quasi-Newton iterations and data mapping. We complement the description of methods for data mapping with a performance and accuracy study using realistic 3D turbine blade meshes. Section 3, in addition, gives details on the installation process of preCICE. Beyond the core of preCICE, we describe the newly-developed, ready-to-use adapters ^20^ for many widely-used simulation software projects in Section 4. Section 5 introduces a simple conjugate heat transfer (CHT) scenario and a simple fluid-structure interaction (FSI) scenario as illustrative examples on how to use preCICE with any of these simulation software projects, followed by the presentation of a systematic multi-level testing infrastructure in Section 6. In the past years, preCICE has become a widely used software ecosystem, for which we have built up a community of users, as we show in Section 7.

Please note, that not all of these sections need to be read at once and neither in the given order. For readers already familiar with preCICE, it is safe to skip Section 3. For readers new to preCICE, it can instead be sufficient to only read Section 3 in the first go, potentially followed by part of Section 4 and Section 5. Section 4 is mainly meant as an overview of all available adapters. It is sufficient to only read those that you intend to use. Finally, Section 6 and Section 7 are rather independent of the rest of the paper.

Our description focuses on (i) usability (by providing robust numerical choices, the multitude of ready-to-use adapter codes, well-structured documentation, and easily-accessible illustrative examples), (ii) reliability (by the systematic multi-level testing concept), and (iii) sustainability (by continuous integration, well-defined development and release cycles and a concept to involve the community in the software development). For a more classical description of preCICE including classical validation with benchmarks, we refer the reader to the first reference paper ^18^. Performance-focused publications ^17, 21^ also demonstrate recent performance and scalability improvements. The contributions of this paper enable new scientific insights in the research fields of our users, but also provide new experiences in scientific software engineering. Results for various applications run with preCICE have been published in many other papers such as ^7– 9, 22, 23^ (see also Section 7).

Naturally, preCICE is not the only general-purpose coupling software that has been developed during the past decades. In the following short summary of related tools, we focus particularly on user-focused and open-source software (i.e., we do not focus on in-house or commercial coupling software, e.g., MpCCI ^24^). There is a number of more multi-scale oriented tools, such as Amuse ^25^, MuMMI ^26^, MUSCLE 3 ^27^, MaMiCo ^28^, or MUI ^29^. Often, the categories of use cases are not strict. MUI, for example, has recently also been used for fluid-structure interaction ^30^. At the same time, current work on preCICE aims towards certain multi-scale coupling patterns (cf. Section 8). A good review on multi-scale coupling software is provided in Groen D, *et al.* ^31^. For climate simulations, a number of specialized tools are available, for example OASIS3-MCT ^32^, YAC ^33^, and C-Coupler2 ^34^. In principle, the term ’coupling’ is not well-defined. For example, software such as pyiron ^35^ or the Kepler Project ^36^ are referred to as *coupling software* as well, whereas they refer to workflow coupling and not a strong coupling between different simultaneously running simulations.

The two software projects most similar to preCICE are presumably DTK ^37^ and OpenPALM ^38^. DTK offers an API that targets lower-level operations compared to preCICE. Its main job is to map and communicate data between different meshes in parallel. The implementation of the actual coupling logic is left to the user, which leads to greater flexibility, but also to more development effort for the user. OpenPALM employs a similar API, provided by CWIPI. The *front end* of OpenPALM, however, provides a higher-level API that includes coupling logic. On top, a graphical user interface is available to configure and steer coupled simulations. The largest difference of preCICE compared to DTK and OpenPALM is presumably the large number of ready-to-use adapters to widely-used simulation software packages (e.g., OpenFOAM, SU2, FEniCS, deal.II, or CalculiX) (cf. Section 4).

## 3 The preCICE library

**Figure 1.**
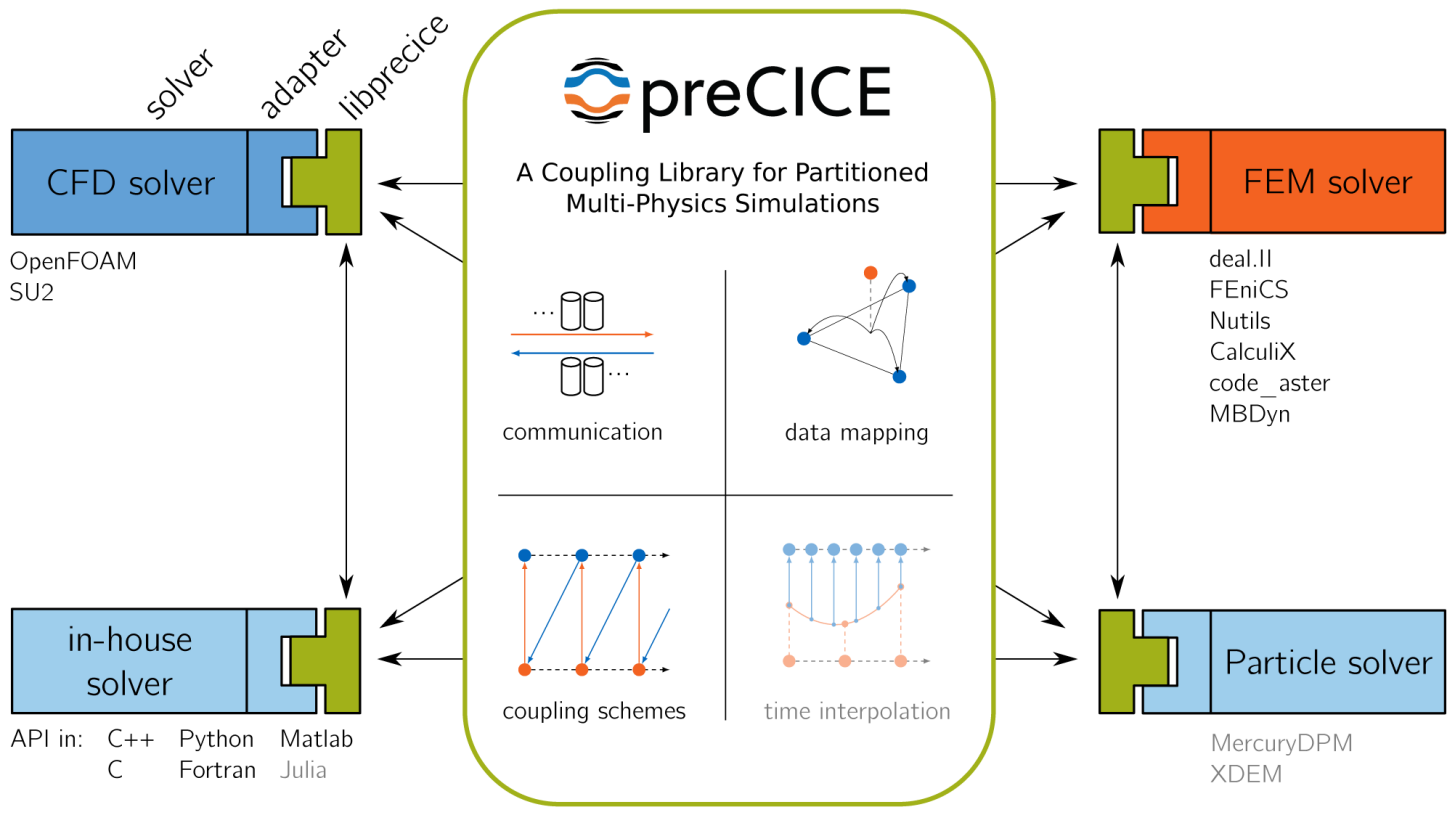
Concept and basic functional components of preCICE. Components that are work in progress and not yet released are shown as faded (time interpolation).

In this section, we present the core library of preCICE in a nutshell. Figure 1 visualizes the concept and basic functional components of preCICE. In the figure, several types of simulation codes are coupled: computational fluid dynamics (CFD) solvers, finite-element method (FEM) solvers, in-house solvers, and particle solvers. Please note that we use these types as examples to introduce the overall concept, not as strict non-overlapping categories. In the following, we refer to coupled simulation codes as *participants* of a coupled simulation. The glue code between a participant’s code and the preCICE library is called adapter. Depending on the participant, an adapter can be a module or a class of the participant’s code or a complete stand-alone software, which uses some callback interface of the participant. In some cases, an adapter can also be a sophisticated script that calls the participant as well as preCICE, but such a software design contradicts the main idea behind the library approach of preCICE to some extent. preCICE comes with several ready-to-use adapters, which are listed in the picture. Not all adapters, though, feature the same level of maturity. Users of preCICE can develop adapters for their own (in-house) codes by using the preCICE API, which is available in many important languages used in scientific computing. An adapter is responsible for what we call *coupling physics*, meaning how to translate nodal coupling values from preCICE into boundary conditions or forcing terms and, reciprocally, how to extract nodal coupling values from internal fields to provide them to preCICE. If we say that an adapter can handle a certain type of coupling physics, for example conjugate-heat transfer, it basically means which type of variables, e.g., temperature or heat flux, the adapter is able to read and write.

preCICE itself has no notion of physics. Instead, preCICE itself is responsible for the technical aspects of coupling and the coupling numerics, depicted in the middle of Figure 1. We now give a first brief overview of these components going from top left to bottom right: (i) Coupled participants are separate executables, potentially running on different nodes in a heterogeneous compute cluster with independent MPI communicators. For instance, a participant running on multiple CPU nodes could be coupled to a participant running on multiple GPU-nodes ^39^. preCICE handles the communication between these executables. The communication is asynchronous and completely parallel. Only those ranks of the participants that need to exchange coupling data communicate with each other. Technically, the communication is based on either MPI Ports or TCP/IP, configurable at runtime; (ii) preCICE implements coupling schemes. Coupling schemes, on the one hand, define the logical coupling flow, i.e., which participant sends which data to which other participant and how the execution of time steps is synchronized between the participants. On the other hand, coupling schemes comprise acceleration methods for implicit coupling such as Aitken under-relaxation or quasi-Newton methods; (iii) Moreover, preCICE allows to map coupling data between non-matching and non-conforming coupling meshes. To this end, the user can choose between projection-based methods (nearest-neighbor or nearest-projection) or radial-basis function interpolation; (iv) Finally, preCICE also handles interpolation in time. Currently, only plain sub-cycling is supported, but higher-order interpolation is under development ^40^ and will be available in future releases.

Please note that, even though Figure 1 depicts a significant green box in the middle, there is no central server-like instance running, even for parallel simulations. preCICE uses a pure peer-to-peer library approach. The only executables that are started are the participants, which all call preCICE.

The current section describes the main concepts of the core library and is structured as follows. In Section 3.1, Section 3.2, and Section 3.3, we describe the methods preCICE uses for coupling schemes, data mapping, and communication, respectively. Different options to get and, if necessary, build preCICE are listed in Section 3.4. Finally, Section 3.5 explains the API and the runtime configuration of preCICE.

### 3.1 Coupling schemes and acceleration

Coupling schemes and acceleration methods are at the very center of the preCICE core and define the coupling flow. As they have been studied in numerous publications (e.g., 41– 43), we restrict the description to a short summary showcasing which combinations of coupling options and acceleration schemes lead to robust and efficient partitioned simulations.

The coupling options can be configured at runtime. preCICE distinguishes: (i) *uni-directional* or *bi-directional* coupling, i.e., data dependencies between the participants in one direction only (example: full flow simulation coupled to an acoustic far field, where the acoustic far fields receives background velocity and pressure values as well as acoustic perturbations at the coupling interface, but we do not observe acoustic waves traveling back into the flow region) or data dependencies in both directions (e.g., fluid-structure interaction); (ii) *explicit* or *implicit* coupling, i.e., execution of each participant once per time step or execution of multiple iterations per time step, such that the values at the end of the time step fulfil all coupling conditions; and (iii) *parallel* or *serial* coupling, i.e., simultaneous or one-after-the-other execution of participants.

**Figure 2.**
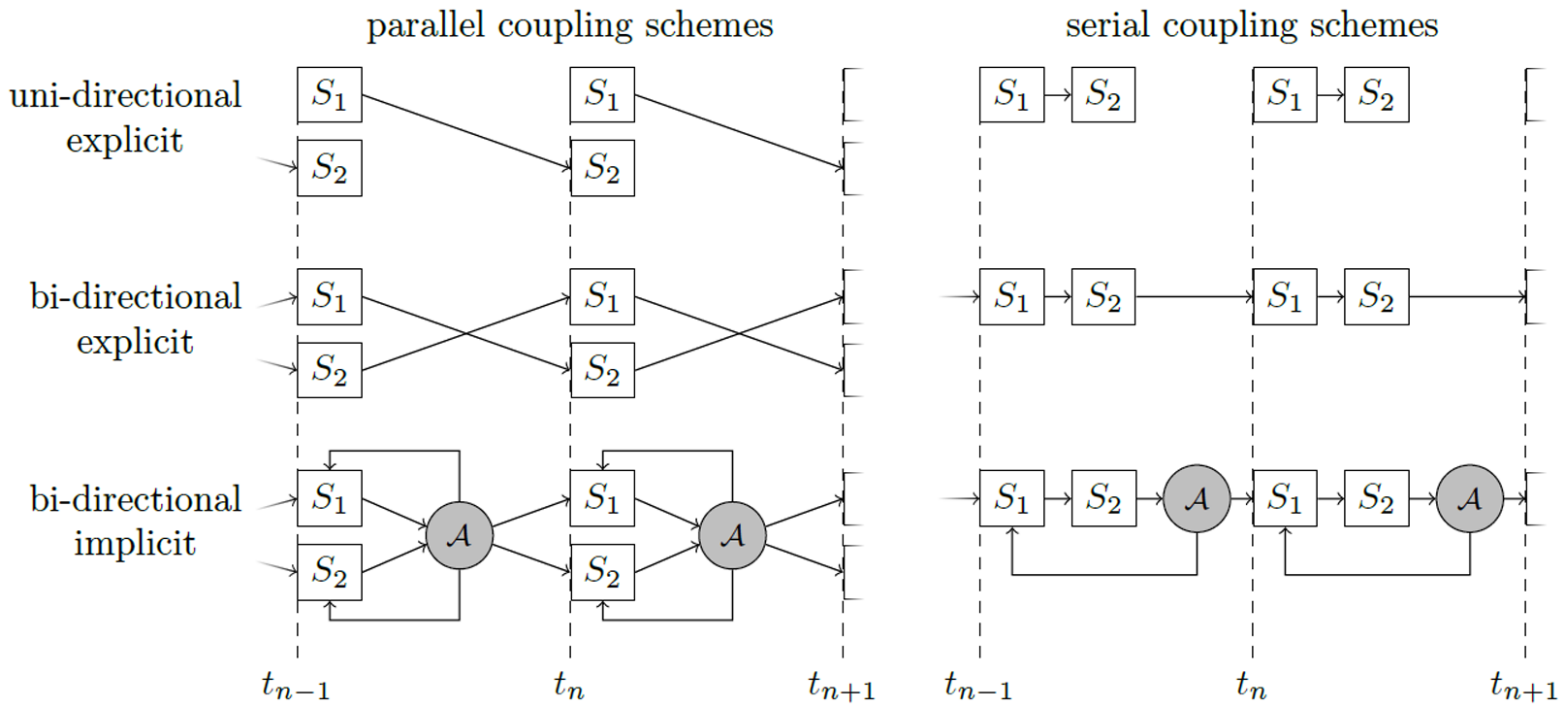
Different coupling options in preCICE for two participants *S*_1_ and *S*_2_ defined by combinations of (i) uni-directional or bi-directional (data transfer between two participants only in one or in both directions); (ii) explicit or implicit (execution of both participants once per time step or iterative solution of a fixed-point equation); (iii) parallel or sequential (simultaneous or one-after-the-other execution of two participants). *A* symbolizes a convergence acceleration method.

Uni-directional coupling requires data transfer only from one participant to the other. Thus, only an explicit coupling makes sense in this case, which can, however, be both serial or parallel. For bi-directional coupling, we have four different coupling scheme options: (i) parallel-explicit, (ii) serial-explicit, (iii) parallel-implicit, (iv) serial-implicit. We show the six different resulting coupling schemes in Figure 2.

In the following, we focus on two non-trivial aspects of coupling: the coupling of more than two participants (multi-code coupling) and the choice of suitable convergence acceleration schemes for implicit coupling.

**Figure 3.**
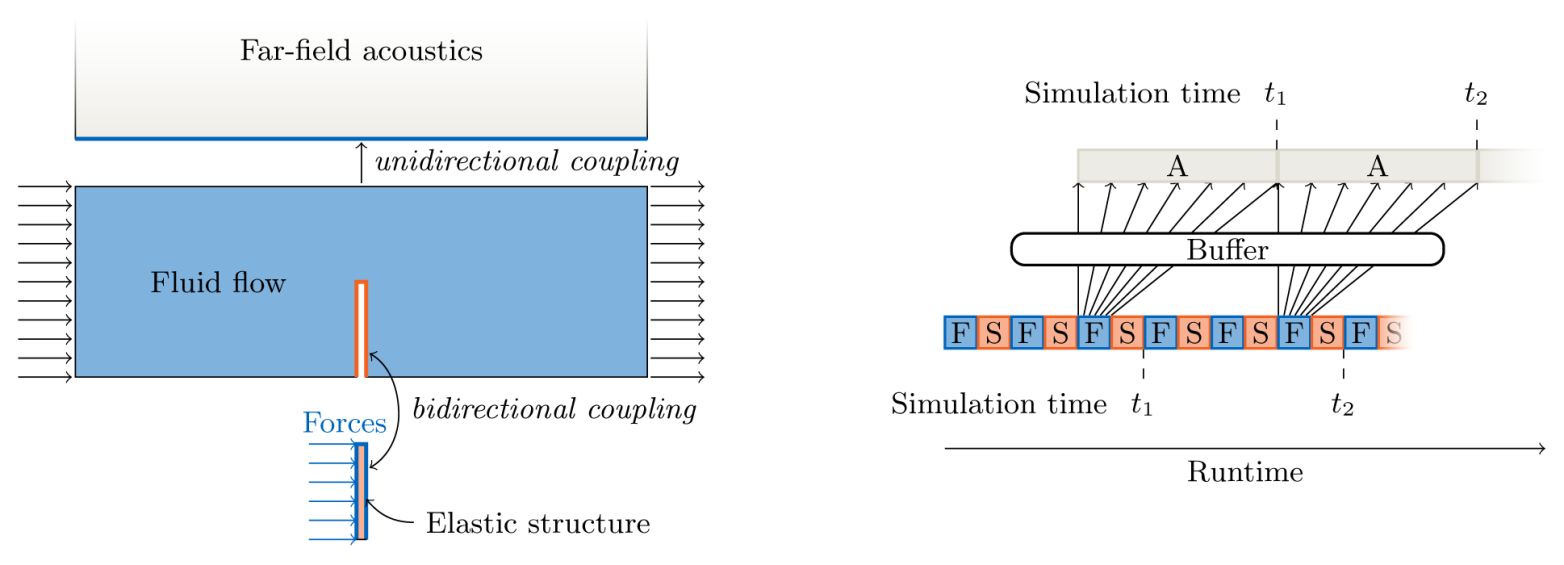
Example for a pairwise multi-code coupling: bi-directional implicit sequential coupling between a structure solver and a flow solver (assuming three iterations per time step) and uni-directional explicit parallel coupling between the flow solver and an acoustics solver. The ExaFSA project report ^6^ presents more details on the required data buffering allowing to achieve parallel efficiency by overlapping the acoustic far field solver with the fluid-structure iterations of the next time step.

**Multi-code coupling** For multi-code coupling, coupling schemes can be configured in two different ways: (i) for each pair of participants separately or (ii) as an overall multi-coupling scheme. For (i), theoretically, all combinations of coupling options are possible. However, combinations of several pairwise implicit coupling schemes have been shown to be numerically unstable ^13^. Still, pairwise coupling can be the best option for some combinations of explicit and implicit coupling. One such example is the extension of the fluid-acoustics example mentioned above ^6^: a bi-directional, implicit, and serial coupling of a structure solver with a flow solver, combined with a uni-directional, explicit, and parallel coupling of the flow solver to the acoustics solver (see Figure 3). To equally balance computational load, this overall scheme requires buffering of the data to be communicated to the acoustics solver, which is another feature provided by preCICE ^6^. In contrast to pairwise coupling, for multi-coupling schemes, the only reasonable realization is parallel coupling, i.e., their combined input and output can be used in the coupling acceleration for implicit coupling as described below.

**Acceleration of implicit coupling iterations** Implicit parts of the coupling schemes described above always require solving a fixed-point equation


H(x)=x.

To give some example, consider serial coupling of two participants *S*_1_ : *x*_1_ ↦ *x*_2_ and *S*_2_ : *x*_2_ ↦ *x*_1_ or the parallel multi-coupling of three participants *S*_1_ : ( *x*_2_, *x*_3_) ↦ ( *y*_2_, *y*_3_), *S*_2_ : *y*_2_ ↦ *x*_3_, and *S*_3_ : *y*_3_ ↦ *x*_3_. The corresponding fixed-point equations read


$$S_{2}\circ S_{1}(x_{1})=x_{1}\;\text{and}\;\{\begin{array}{ccc} S_{1}(x_{2,}x_{3}) & = & (y_{2,}y_{3}),\\ S_{2}(y_{2}) & = & x_{2},\\ S_{3}(y_{3}) & = & x_{3}. \end{array}\}$$

If multiple coupling data vectors are combined in a single fixed-point equation as in the last example, it is numerically beneficial to bring all data to the same scale by an automatic weighting (called *preconditioner* in preCICE ^15^).

The pure fixed-point iterations can be enhanced with an accelerator *A*, which uses all input and output information of the operator *H* collected in previous iterations:


$$x^{k+1}=A(x^{0},\;.\;.\;.\;,x^{k},H(x^{0}),\;.\;.\;.\;,H(x^{k})).$$

The simplest acceleration scheme is Aitken’s under-relaxation as presented, e.g., by Küttler *et al*. ^44^. It reuses only information from the last iteration. For most applications, quasi-Newton schemes


$$A(x^{0},\;.\;.\;.\;,x^{k},H(x^{0}),\;.\;.\;.\;,H(x^{k}))={\tilde{x}}^{k}+(W_{k}-J^{\text{prev}}V_{k})\;\alpha^{k}-J^{\text{prev}}R(x^{k})\;(1)$$

are significantly more efficient and robust ^15^. Here, *α ^k^* is a coefficient vector, $\tilde{x}$ *^k^* ≔ *H*( *x ^k^*), *R*( *x ^k^*) ≔ *H*( *x ^k^*) − *x ^k^*. We use the matrix *J* ^prev^ to include knowledge about the inverse Jacobian already achieved in previous time steps ^45^. In the classical quasi-Newton methods for fluid-structure interaction as introduced by Degroote *et al*. ^46^, *J* ^prev^ is zero. The matrices *V _k_* and *W _k_* collect residual and value differences throughout previous iterations:


$$\begin{array}{l} W_{k}=[\text{Δ}{\tilde{x}}_{0}^{k},\text{Δ}{\tilde{x}}_{1}^{k},\;.\;.\;.\;,\text{Δ}{\tilde{x}}_{k-1}^{k}],\;\text{with}\;\text{Δ}{\tilde{x}}_{i}^{k}={\tilde{x}}^{k}-{\tilde{x}}^{i},\\ \;V_{k}=\;\;[\text{Δ}r_{0}^{k},\text{Δ}r_{1}^{k},\;.\;.\;.\;,\text{Δ}r_{k-1}^{k}],\;\text{with}\;\text{Δ}r_{i}^{k}=R(x^{k})-R(x^{i}) \end{array}$$

with the number *k* of iterations done so far ^1^. In practice, the leftmost columns of *V _k_* and *W _k_* can always be dropped in cases where several iterations ( *k*) are required for convergence.

Equation (1) is an approximation of the modified Newton iteration


$$x^{k+1}={\tilde{x}}^{k}-J_{\tilde{R}}^{-1}R(x^{k}),$$

where $J_{\tilde{R}}^{-1}$ is the inverse Jacobian of $\tilde{R}$ : $\tilde{x}$ *^i^* → *R*( *x ^i^*). To derive Equation (1), $J_{\tilde{R}}^{-1}$ is approximated by the solution $J_{k}^{-1}$ = ( *W _k_* – *J* ^prev^ *V _k_*) $V_{k}^{T}$ ( *V _k_* $V_{k}^{T}$ ) ^–1^ + *J* ^prev^ of the multi-secant equation ^2^


$$J_{k}^{-1}V^{k}=W^{k}\;\text{under}\;\text{the}\;\text{norm}\;\text{minimization}\;{\left\|J_{k}^{-1}-J^{\text{prev}}\right\|}_{F}\leftarrow \text{min},\;(2)$$

where ‖ · ‖ *_F_* denotes the Frobenius norm. In the following, we shortly present the main two quasi-Newton classes, IQN-ILS and IQN-IMVJ, provided by preCICE, and we introduce the so-called filtering that can improve the robustness of both.

**IQN-ILS** For *J* ^prev^ = 0, we get the classical *interface quasi-Newton inverse least squares* method as introduced by Degroote *et al*. ^46^. For this approach, re-using information from previous time steps by adding further columns to *V _k_* and *W _k_* can help speed up the coupling iterations significantly. However, the optimal number of reused time steps strongly depends on the involved equations, on the discretization of the respective fields and even their mesh resolution ^43^.

**IQN-IMVJ** For *J* ^prev^ chosen as the last inverse Jacobian approximation of the previous time step, an idea adopted from the work of Bogaers *et al*. ^45^ in later studies ^42, 47^, we get the method called *interface quasi-Newton inverse multi-vector Jacobian*. A variety of restart mechanisms allows us to implement this method with linear complexity in the number of coupling unknowns by avoiding storing the full matrix *J* ^prev^. Only low-rank additive components are stored and the respective sum is re-set after a chunk of time steps. The size of these chunks is configurable by the user, but its influence on the convergence behavior is not very significant ^43, 48^.

**Filtering ** Since linear dependencies of columns in *V _k_* cannot be avoided in both IQN-ILS and IQN-IMVJ, we implemented various filtering algorithms, which automatically delete columns that cause (near) linear dependencies. This eliminates both contradicting and outdated information ^49^.

All quasi-Newton variants are implemented in a fully-parallel way based on parallel *QR*-solvers for the calculation of components of $J_{k}^{-1}$ and the matrix-vector product $J_{k}^{-1}$ *R*( *x ^k^*). We do not present numerical results in this section, but refer to separate publications ^15, 41, 43^ for detailed comparisons of quasi-Newton variants and examples showcasing their efficiency and robustness.

### 3.2 Data mapping

In a coupled simulation, participants exchange data via coupling meshes. The coupling meshes of each pair of participants discretize either the common coupling interface (for surface coupling) or a common volume (for volume coupling). However, the discretization approaches of the two participants are usually different, leading to non-matching meshes. In order to transfer physical variables between these non-matching meshes, we use data mappings.

In the current version of preCICE, provided data mapping methods are restricted to methods that work in a black-box way on point clouds (nearest-neighbour mapping, radial basis function interpolation) or with a minimum of additional information (nearest-projection mapping). A flexibilization of the mapping concept to facilitate also customized mapping, e.g., for high order spatial discretizations, is work in progress and will be provided in future versions.

In the following subsection, we shortly describe the types of data mapping implemented in preCICE and give some results showcasing their performance and accuracy.

Let the dimension of the scenario *d* be two or three and let us consider a data mapping from the coupling mesh of participant *S*_1_ to the coupling mesh of participant *S*_2_. The minimum mesh information required (during initialization) is the vertex coordinates of both meshes, which we define as:


$$M_{S_{1}}=\{{\vec{x}}_{1}^{S_{1}},\;.\;.\;.\;,\;{\vec{x}}_{n_{1}}^{S_{1}}\}\;\text{with}\;{\vec{x}}_{i}^{S_{1}}\in {\mathbb{R}}^{d},\;M_{S_{2}}=\{{\vec{x}}_{1}^{S_{2}},\;.\;.\;.\;,\;{\vec{x}}_{n_{2}}^{S_{2}}\}\;\text{with}\;{\vec{x}}_{i}^{S_{2}}\in {\mathbb{R}}^{d}.$$

Data mapping aims to map the vector ${\vec{v}}^{S_{1}}={\left(v_{1}^{S_{1}},v_{2}^{S1},\;.\;.\;.\;,v_{n_{1}}^{S_{1}}\right)}^{T}$ of values at the vertices in *M*_*S*_1__ to the vector ${\vec{v}}^{S_{2}}={\left(v_{1}^{S_{2}},v_{2}^{S_{2}},\;.\;.\;.\;,v_{n_{2}}^{S_{2}}\right)}^{T}$ of values at the vertices in *M*_*S*_2__.

All data mapping methods in preCICE are provided in a consistent and in a conservative variant. Consistent mapping operations exactly reproduce constant data at *M*_*S*_1__ on *M*_*S*_2__. Conservative mapping methods preserve the sum of all values. Consistent mapping schemes are, thus, used for physical variables such as displacements, velocities, pressure, or stresses, whereas conservative methods have to be used for cumulative variables, such as forces. Note that higher-order consistency or more sophisticated conservation properties like conservation of integral values in the particular higher-order finite element basis of a solver is currently not feasible due to the black-box character of the mapping. However, the above-mentioned ongoing flexibilization is going to help tackle this restriction as well.

Data mapping can be written as a linear mapping


$$M{\vec{v}}^{S_{1}}={\vec{v}}^{S_{2}}$$

with a matrix *M* ∈ ℝ ^*n*_2_ ×^ ^*n*_1_^. For a consistent mapping, the sum of entries in each row of the mapping matrix *M* has to be one, whereas, for a conservative mapping, the requirement is $\sum_{i}v_{i}^{S_{1}}=\sum_{i}v_{i}^{S_{2}}$ , and thus, the sum of entries in each column of *M* has to be one. Therefore, conservative mapping methods are generated by transposing the mapping matrix of a consistent mapping. Throughout this section, we restrict our explanation to consistent data mapping. For details on both variants, the reader is referred to mapping-focused publications ^14, 16, 50^.

***3.2.1 Projection-based data mapping.*** Two projection-based mappings are available in preCICE: nearest-neighbor and nearest-projection. The nearest-neighbor mapping establishes an association between each vertex ${\vec{x}}_{i}^{S_{2}}$ of the output mesh *M*_*S*_2__, with the spatially nearest vertex ${\vec{x}}_{j(i)}^{S_{1}}$ on the input mesh *M*_*S*_1__.

The mapping is then simply defined as


$$v_{i}^{S_{2}}=v_{j(i)}^{S_{1}}.$$

**Figure 4.**
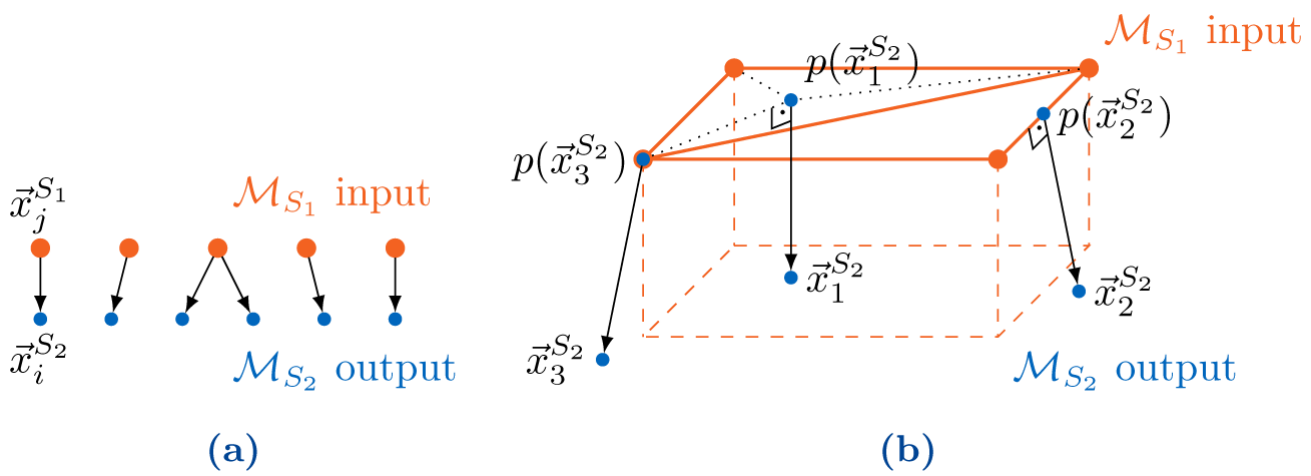
Basic principles of nearest-neighbor and nearest-projection mapping: ( **a**) Transfer of each value $v_{j(i)}^{S_{1}}$ at the nearest neighbor ${\vec{x}}_{j(i)}^{S_{1}}$ in the coupling mesh of *S*_1_ to the vertex ${\vec{x}}_{i}^{S_{2}}$ in the coupling mesh of *S*_2_. ( **b**) Projection of points ${\vec{x}}_{1}^{S_{2}}$ , ${\vec{x}}_{2}^{S_{2}}$ , and ${\vec{x}}_{3}^{S_{2}}$ of the coupling mesh of *S*_2_ to a triangle, an edge, and a vertex, respectively, of the coupling mesh of *S*_1_.

The nearest-projection mapping uses connectivity information between multiple vertices on the input mesh to interpolate to a vertex on the output mesh. To calculate the value at an output vertex ${\vec{x}}_{j}^{S_{2}}$ , we calculate a projection point $p\left({\vec{x}}_{j}^{S_{2}}\right)$ on the entities of the input mesh, interpolate a value to this projection point, and copy this to the output vertex ${\vec{x}}_{j}^{S_{2}}$ . The projection point $p\left({\vec{x}}_{j}^{S_{2}}\right)$ is a projection on a triangle of the input mesh defined by participant *S*_1_. If such a triangle does not exist, we determine $p\left({\vec{x}}_{j}^{S_{2}}\right)$ via orthogonal projection to an edge in the coupling mesh of participant *S*_1_ or, as the last option, as the closest vertex in the mesh of participant *S*_1_. This requires a search operation over triangles and potentially edges and vertices of the mesh of participant *S*_1_ for each output vertex ${\vec{x}}_{j}^{S_{2}}$ . See Figure 4 for the relation between mesh entities of the two meshes. In the second step of the mapping, we use barycentric interpolation (if $p\left({\vec{x}}_{j}^{S_{2}}\right)$ is in a triangle), linear interpolation (if $p\left({\vec{x}}_{j}^{S_{2}}\right)$ is on an edge) or the respective vertex value (if $p\left({\vec{x}}_{j}^{S_{2}}\right)$ is a vertex) to determine a value at $p\left({\vec{x}}_{j}^{S_{2}}\right)$ and then use this value as $v_{j}^{S_{2}}$ at the output point. In other words, the interpolation is a combination of a second-order accurate interpolation inside a triangle in *S*_1_ and a first-order accurate extrapolation in normal direction, i.e., the error is ( *h* ^2^) + ( *δ*) with the mesh width *h* of the mesh of *S*_1_ and the normal distance δ between the two coupling meshes. In cases where the mesh of participant *S*_1_ does not provide suitable mesh connectivity information, a simple projection onto the closest vertex is performed, i.e., the nearest-projection mapping falls back to nearest-neighbor.

Both nearest-neighbor and nearest-projection mapping require neighbor search between mesh entities of both participants. To implement this search efficiently, we generate r-start index-trees for vertices, edges, and triangles of meshes using the Geometry package of Boost ^3^. The complexity of generating the index tree is *O*( *n* log( *n*)) and the complexity of each nearest neighbor query is *O*(log( *n*)) if *n* is the number of entities in the involved meshes.

**Figure 5.**
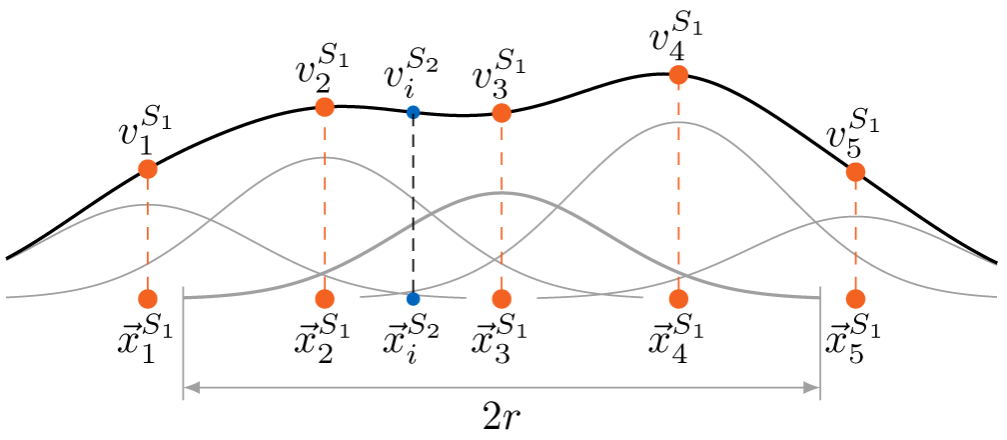
Simplified one-dimensional view of the generation of the interpolant and the evaluation at the target point ${\vec{x}}_{i}^{S_{2}}$ based on a linear combination of Gaussian basis functions with support radius *r*, neglecting the global polynomial.

***3.2.2 Data mapping with radial basis functions.*** Radial basis function (RBF) mapping uses a linear combination of radially symmetric basis functions centered at vertices ${\vec{x}}_{i}^{S_{1}}$ of the input mesh to create a global interpolation function, which is afterwards sampled at the vertices of the output mesh ${\vec{x}}_{i}^{S_{1}}$ . In order to ensure that constant and linear functions are interpolated exactly, an additional global first-order polynomial term *q*( $\vec{x}$ ) is added to the interpolant *s* : ℝ ^*d*^ → ℝ:


$$s(\vec{x})=\sum_{i=1}^{n_{1}}{\text{λ}}_{i}\;\cdot \;\phi \left(\|\vec{x}-{\vec{x}}_{i}^{S_{1}}{\|}_{2}\right)+q(\vec{x}),$$

where the radial basis function is given by *ϕ*, and the polynomial term *q* ( $\vec{x}$ ) = *β*_0_ + *β*_1_ *x*_1_ + . . . + *β* *_d_* *x _d_*. Several basis functions available in preCICE are listed in Table 1. See Figure 5 for a schematic view of the relation between the vertices of both coupling meshes and the construction and evaluation of the interpolant.

**Table 1.** Radial basis functions available in preCICE (excerpt). Local basis functions have a support radius *r*, i.e., *ϕ* ( $\left||\vec{x}|\right|$ _2_) = 0 for $\left||\vec{x}|\right|$ _2_ > *r*. C-TPS use normalized variables ξ = $\left||\vec{x}|\right|$ _2_/ *r* and are set to zero for *ξ* > 1. We enforce a finite support radius for Gaussians by setting the basis function to zero when falling below a threshold of 10 ^−9^ ^51^. For a given support *r*, we can, thus, compute the necessary shape parameter *ζ*.

	Basis Function	Support
Gaussians	exp(−(ζ· $\left||\vec{x}|\right|$ ) ^2^)	Local
Global Thin Plate Splines (G-TPS)	$\left||\vec{x}|\right|$ ^2^ log( $\left||\vec{x}|\right|$ )	Global
Compact Thin Plate Splines C2 (C-TPS)	1 − 30ξ ^2^ − 10ξ ^3^ + 45ξ ^4^ − 6ξ ^5^ − 60ξ ^3^ log ξ	Local

The set of coefficients λ _*i*_ ∈ ℝ, *i* = 1, ..., *n*_*S*_1__ is determined such that the interpolation conditions


$$s\left({\vec{x}}_{i}^{S_{1}}\right)=v_{i}^{S_{1}}\;\forall i=1,\ldots ,n_{1}$$

are fulfilled. The addition of the polynomial term leads to an under-determined system which is regularized by the polynomial conditions


$$\sum_{i=1}^{n_{S_{1}}}{\text{λ}}_{i}\;\cdot {\vec{x}}_{i}^{S_{1}}=0\;\text{and}\;\sum_{i=1}^{n_{S_{1}}}{\text{λ}}_{i}\;=0.$$

In matrix notation, this leads to the linear system


$$\left(\begin{array}{c} C\\ Q^{T} \end{array}\;\begin{array}{c} Q\\ 0 \end{array}\right)\;\left(\begin{array}{c} \vec{\text{λ}}\\ \vec{\beta } \end{array}\right)=\left(\begin{array}{c} {\vec{v}}^{S_{1}}\\ \vec{0} \end{array}\right),$$

where $\vec{\lambda }$ = (λ _1_, λ _2_, . . . , λ _*n*_1__) ^*T*^, *β* = ( *β*_0_, *β*_1_, . . . , *β*_*d*_) ^*T*^, $C={\left(\phi \left(||{\vec{x}}_{i}^{S_{1}}-{\vec{x}}_{j}^{S_{1}}|{|}_{2}\right)\right)}_{i,j=1,\ldots ,n_{1}}$ ∈ ℝ ^*n*_1_ × *n*_1_^,

and $Q={\left(1,\;x_{1,i}^{S_{1}},\ldots ,\;x_{d,i}^{S_{1}}\right)}_{i=1,\ldots ,n_{1}}$ ∈ ℝ ^*n*_1_ × ( *d*+1)^

and, finally, the mapping reads


$${\vec{v}}^{S_{2}}=\left(\tilde{C}\;\tilde{Q}\right)\;{\left(\begin{array}{c} C\\ Q^{T} \end{array}\;\begin{array}{c} Q\\ 0 \end{array}\right)}^{-1}\;\left(\begin{array}{c} {\vec{v}}^{S_{1}}\\ \vec{0} \end{array}\right)$$

with $\tilde{C}={\left(\phi \left(||{\vec{x}}_{i}^{S_{2}}-{\vec{x}}_{j}^{S_{1}}|{|}_{2}\right)\right)}_{\begin{array}{l} i=1,\ldots ,n_{2}\\ j=1,\ldots ,n_{1} \end{array}}$ ∈ ℝ ^*n*_2_^ × ^*n*_1_^,

$\tilde{Q}={\left(1,\;x_{1,i}^{S_{2}},\;x_{2,i}^{S_{2}},\;x_{3,i}^{S_{2}}\right)}_{i=1,\ldots ,n_{2}}$ ∈ ℝ ^*n*_2_ × 4^.

Local basis functions result in a sparse matrix *C*. However, the polynomial term matrix *Q* is always densely populated, which hampers the favorable properties of the sparse matrix. Solving the polynomial term separately in a least squares approach via QR-decomposition of the matrix *Q* capitalizes on the sparsity of the matrix *C*. For a full description of this separated polynomial approach, a separate publication is available ^51^.

As radial basis functions are radially symmetric in all spatial dimensions, distances between the two involved coupling meshes normal to a coupling surface do not have to be explicitly tackled, in contrast to the nearest-projection method. However, the accuracy of the RBF mapping decreases with an increasing gap or overlap between the two meshes of *S*_1_ and *S*_2_. In addition, the RBF mapping with local basis functions suffers from a trade-off between high accuracy (achieved for basis functions with wide support) and feasible conditioning of the linear system (only given for moderate support width). We address the latter to some extent by scaling the interpolant with the interpolant of the constant unit function, which allows us to use a smaller support radius without deteriorating accuracy ^51, 52^.

The RBF data mapping is implemented using either (depending on configuration) an iterative generalized minimal residual method (GMRES) solver from PETSc ^53^ in every mapping step, or an initial dense QR-decomposition from Eigen ^54^ followed by a matrix-vector product and a backward substitution in every mapping step. While the GMRES solver is fully parallelized, the QR-decomposition uses a sequential computation on a single rank.

The RBF mapping as described here is consistent, i.e., exactly reproduces constant input if the interpolation problem is solved exactly. In this case, the constant part of the polynomial is the exact and correct solution. Otherwise, we get consistency only up the solver accuracy of the GMRES solver. Similar arguments hold for the conservative variant which is realized by a formal transposition of the mapping matrix translated into respective solver steps ^16^.

***3.2.3 Numerical and runtime performance.*** We compare the various data mapping methods in terms of accuracy and computational demand using the Artificial Solver Testing Environment (ASTE) ^4^. ASTE imitates data input and output of participants coupled via preCICE in an artificial setting. In our test setup, two ASTE participants, *S*_1_ and *S*_2_, are coupled via preCICE. Both define individual surface meshes of the same geometry. We then use an analytical test function,


$$f(\vec{x})=0.78+\text{cos}(10\cdot (x_{1}+x_{2}+x_{3}))\;,$$

to set values on *M*_*S*_1__. We compute a single consistent mapping from *M*_*S*_1__ to *M*_*S*_2__ and measure errors on *M*_*S*_2__ with a discrete *l*_2_-norm, 


$${\left(\frac{1}{n}\sum_{i=1}^{n}{\left(v_{i}^{S_{2}}-f({\vec{x}}_{i}^{S_{2}})\right)}^{2}\right)}^{\frac{1}{2}}.$$

**Figure 6.**
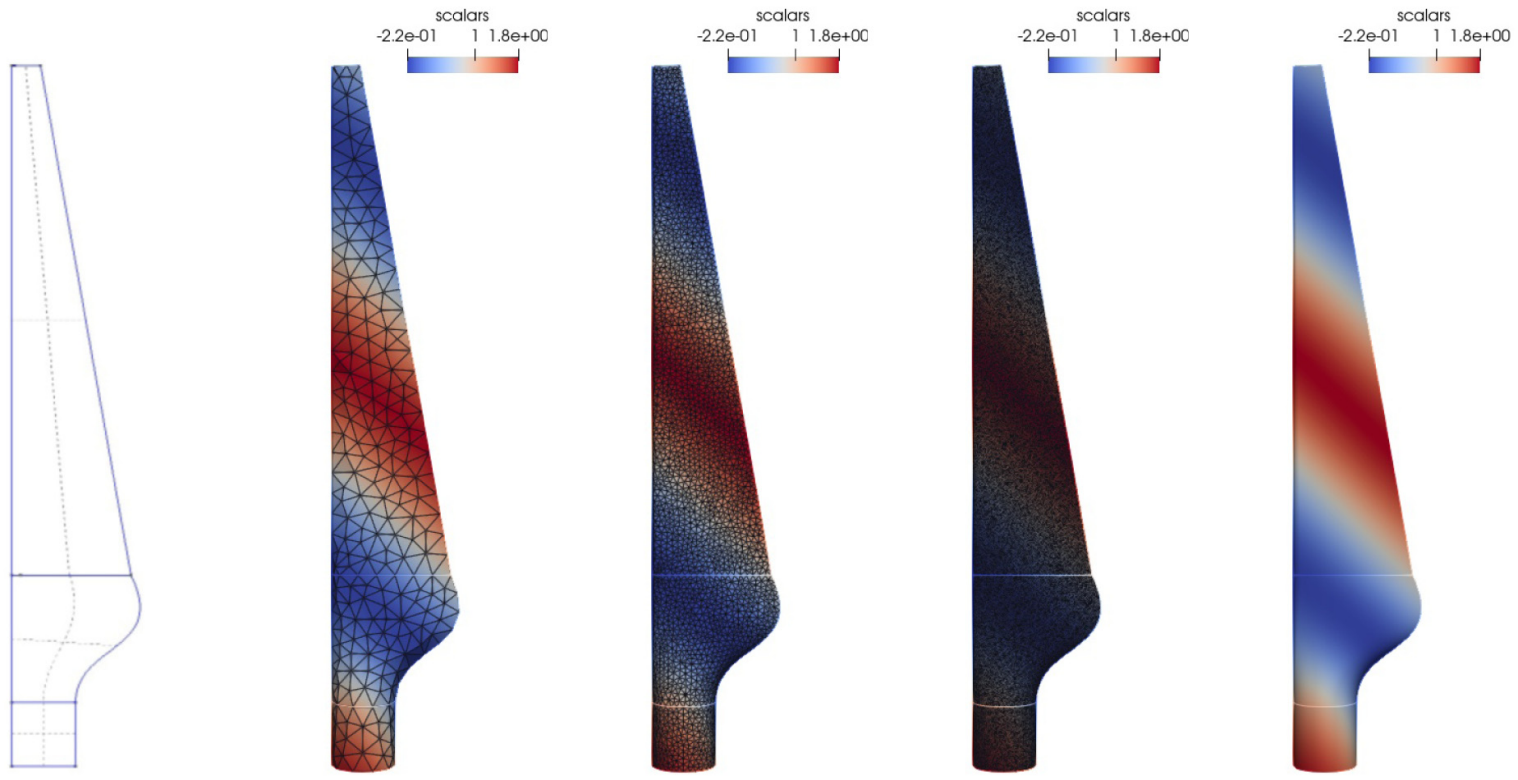
Mapping test case: Different meshes of the turbine-blade test geometry. From left to right: the geometry used to generate the meshes, *h* = 0.03, *h* = 0.009, *h* = 0.004, *h* = 0.0005 without edges. The mesh surface color indicates the test function, edges are drawn in black.

As test geometry, we use a wind turbine blade ^5^. We use GMSH ^55^ to generate almost uniform surface meshes with different resolutions as listed in Table 2. In Figure 6, we visualize the geometry, different meshes, and the test function.

**Table 2.** The meshes used for the mapping tests sorted from coarse to fine. Bold typesetting indicates the output meshes (associated to participant S _2_). All other meshes are used as input meshes (associated to participant S _1_).

*h*	Vertices	Triangles	Series
0.03	438	1007	coarse
0.02	924	2027	coarse
0.01	3458	7246	coarse
0.009	4302	8970	**coarse**
0.008	5310	11025	coarse
0.006	9588	19712	coarse
0.004	21283	43352	fine, coarse
0.003	38112	77271	fine
0.002	84882	171319	fine
0.0014	172803	347815	**fine**
0.001	338992	681069	fine
0.0007	691426	1387249	fine
0.0005	1354274	2714699	fine

We run the mapping tests on the thin-nodes partition of SuperMUC-NG, hosted at the Leibniz Supercomputing Centre. Each thin-node contains two 3.1GHz Intel Xeon Platinum 8174 (SkyLake) processors with a total of 48 cores and 96GB of system memory per node. The tests used the Intel Omni-Path interconnect as primary network connection. We run participant *S*_1_ on a single node (48 MPI ranks) and participant *S*_2_ on two nodes (96 MPI ranks) as participant *S*_2_ computes the actual mapping. Runtime is the maximum of a given event over all ranks of *S*_2_^6^ and memory is the sum of the peak memory usage of all ranks of *S*_2_. These results are in addition averaged over five runs. For the RBF data mapping variants, we use GMRES as a linear solver with a relative convergence threshold of 10 ^−6^, except for G-TPS, where we use the sequential QR-decomposition.

We compare the data mapping methods in two series of computations. The first, *coarse* series is based on meshes with *h* ≥ 0.004, where participant *S*_2_ always uses *h* = 0.009 and participant *S*_1_ varies the value of *h* through all other values of the series listed in Table 2. The second, *fine* series is based on meshes with *h* ≤ 0.004. Participant *S*_2_ uses *h* = 0.00014 and participant *S*_1_ the rest. While we can compare all mapping variants for the coarse series, RBF data mapping with G-TPS is too expensive in terms of computation and memory for the fine series. For reproduction of our results, all data used as well as all steps are available in a data repository ^56^.

**Figure 7.**
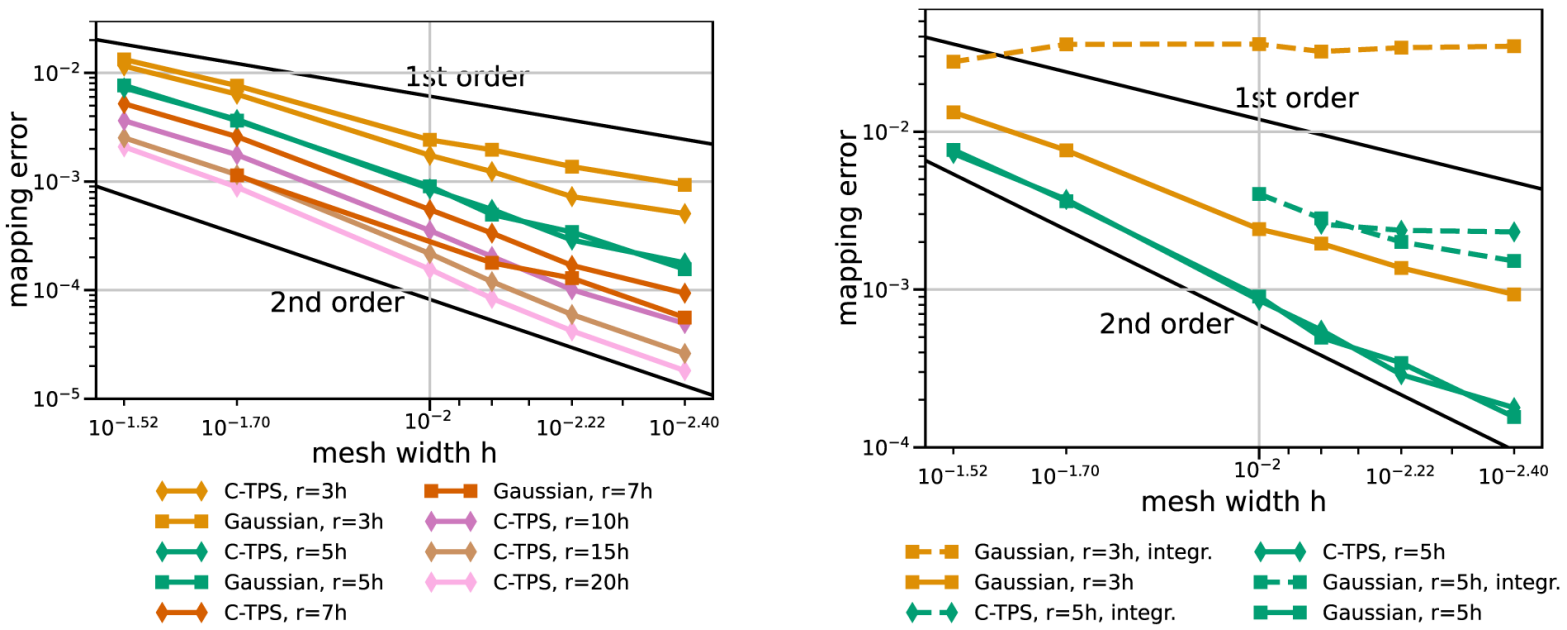
RBF data mapping with local basis functions for coarse series. Comparison of various support radii (left) and integrated and separated handling of the global polynomial (right). Participant *S*_2_ uses *h* = 0.009. Missing data points mark diverging cases. Gaussians with *r* = 7 *h* or *r* = 10 *h* diverge for some or all cases, respectively (left). With integrated handling of the polynomial, both basis functions diverge for *r* = 5 *h* and coarser meshes (right).

Figure 7 gives results for RBF data mapping with local basis functions – C-TPS and Gaussians – for varying support radii and for integrated and separated handling of the global polynomial. Separated handling of the polynomial clearly outperforms integrated handling in terms of accuracy and robustness. For C-TPS, an increase in accuracy is observed for increasing support radius. This is even true for rather large radii ( *r* = 20 *h*), where we get more than quadratic convergence. Gaussians, however, show robustness issues for larger support radii. We assume that this behavior is caused by the increasing ratio of large to small matrix entries. In a further test (not shown here), we observed that increasing the (hard-coded) threshold value from 10 ^−9^ to 10 ^−5^ seems to improve robustness.

**Figure 8.**
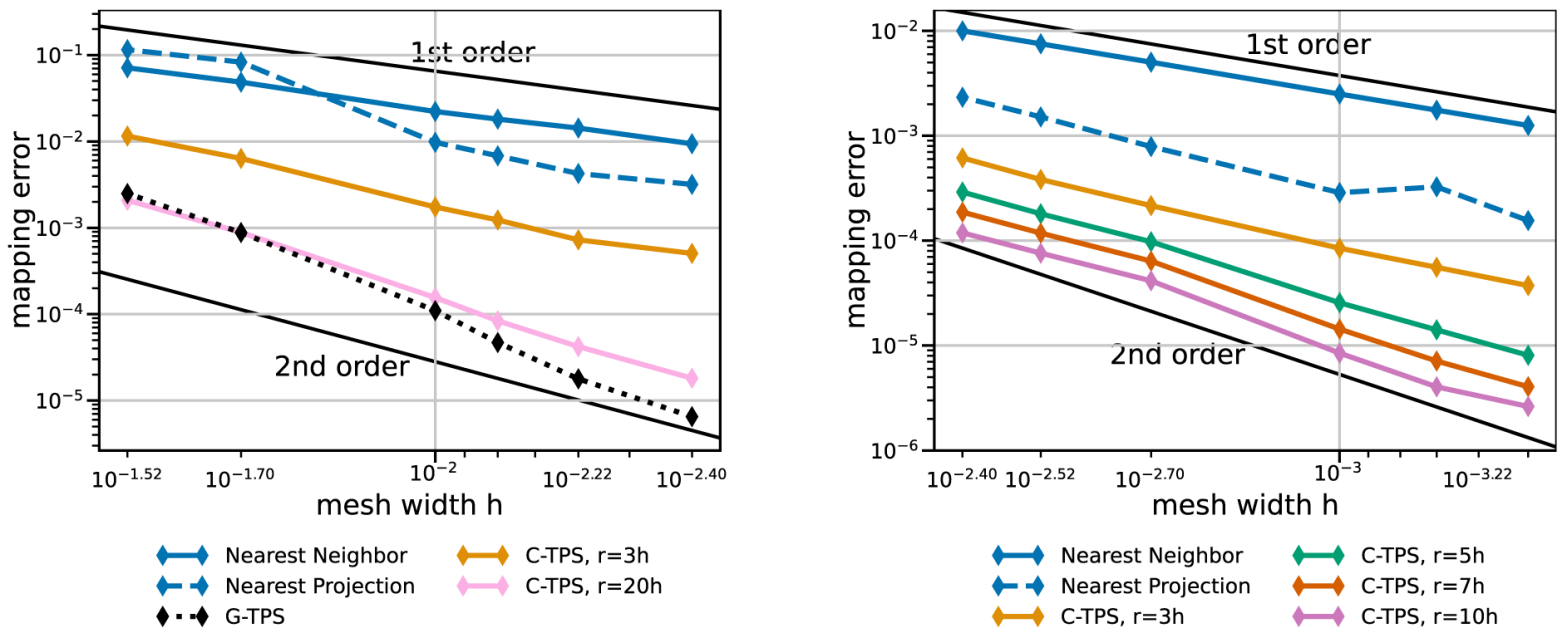
Comparison of nearest-neighbor / nearest-projection mapping and RBF mapping with G-TPS and C-TPS for coarse series (left) and fine series (right) from Table 2. RBF mapping with G-TPS is infeasibly expensive for the fine series. All RBF mapping methods use a separated handling of the polynomial. Participant *S*_2_ uses *h* = 0.009 for the coarse series (left) and *h* = 0.0014 for the fine series (right).

Figure 8 sets the best local RBF variants in perspective with RBF mapping using G-TPS, nearest-neighbor mapping, and nearest-project mapping. RBF mapping using C-TPS with a large support radius is comparable to RBF mapping using G-TPS. The latter only wins for the finest meshes. RBF data mappings clearly outperform nearest-neighbor and nearest-projection mapping – even for a relatively small support radius of *h* = 3 *r*. Nearest-projection mapping, interestingly, does not show a constant second-order convergence for the coarse series, which suggests that the projection error dominates the interpolation error. In fact, similar tests with a cubic geometry (not shown) give constant second-order convergence for nearest-projection mapping as, in this case, all meshes directly lie on the geometry (i.e. no projection error). For the fine series, nearest-projection mapping gives a rather constant second-order convergence as well.

**Figure 9.**
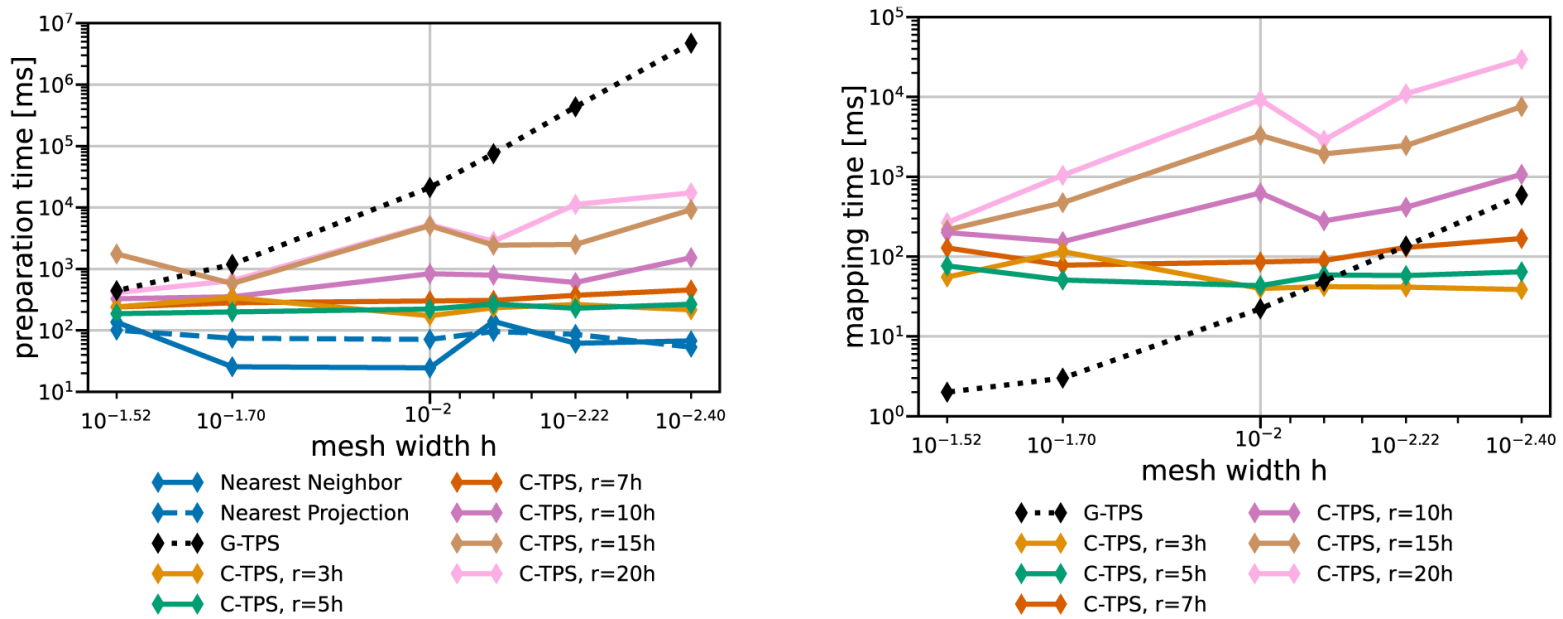
Comparison of one-time preparation time (left) and the recurrent mapping time (right) of various data mapping methods for the coarse series from Table 2. All RBF mapping methods use a separated handling of the polynomial. The recurrent mapping time of nearest-projection and nearest-neighbor mapping is below the measurement resolution of 1 *ms* and hence omitted. Participant *S*_2_ uses *h* = 0.009.

**Figure 10.**
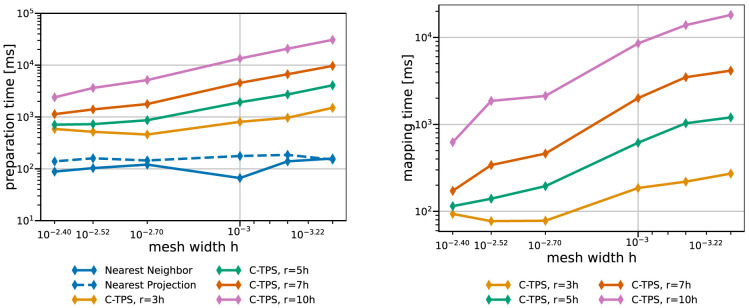
Comparison of one-time preparation time (left) and the recurrent mapping time (right) of various data mapping methods for the fine series. All RBF mapping methods use a separated handling of the polynomial. The one-time preparation of the nearest-neighbor mapping is an inexpensive operation and has the tendency to fluctuate, five samples are not enough to fully smooth them out resulting in a spike at 0.001. The recurrent mapping time of nearest-projection and nearest-neighbor mapping is below the measurement resolution of 1 *ms* and hence omitted. Participant *S*_2_ uses *h* = 0.0014.

**Figure 11.**
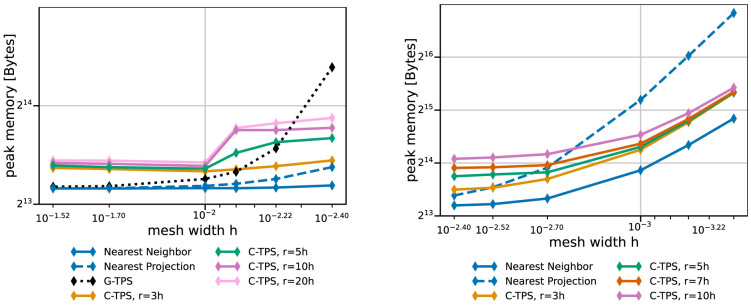
Comparison of the maximum overall memory consumption of various data mapping methods for coarse series (left) and fine series (right) from Table 2. Participant *S*_2_ uses *h* = 0.009 for the coarse series (left) and *h* = 0.0014 for the fine series (right). The memory consumption is the maximum of all ranks of participant *S*_2_, which is executing the mapping.

Next, we compare the same methods in terms of compute time. Here, we have to distinguish between one-time preparation time (e.g., QR-factorization, matrix initialization in PETSc, or nearest-neighbor search) and recurrent mapping time in every mapping operation (e.g., back substitution or GMRES solve). Figure 9 and Figure 10 give both data for various data mapping methods and the coarse and fine series, respectively. The quickly increasing preparation time of RBF mapping with G-TPS makes the method unpractical for finer meshes. For C-TPS, both the preparation time and the recurrent mapping time increases significantly with increasing support radius and decreasing mesh width. For fine meshes, larger support radii are thus discouraged despite their superior accuracy. For small cases, the overhead of the PETSc solver for RBF is more costly than the overall serial QR-factorization in G-TPS. Nearest-neighbor and nearest-projection mapping are both drastically cheaper than RBF data mapping, particularly in the recurrent mapping time. Finally, Figure 11 compares the peak memory consumption of all data mappings. RBF mapping with G-TPS shows a drastic increase in memory with increasing mesh size. For the coarse series, all methods show the expected behavior: higher memory consumption for RBF than for nearest-neighbor and nearest-projection mapping and increasing memory consumption for RBF mapping using C-TPS with increasing support radius. For the fine series, the nearest-project surpassed even the RBF methods, due to the additional cost of handling connectivity information.

We conclude that RBF data mapping with local basis functions is a useful method. There is a natural trade-off between accuracy and compute effort when modifying the support radius. A good compromise is a support radius of *r* = 5 *h* to *r* = 7 *h*. In the current implementation, C-TPS should be preferred over Gaussians as basis functions. RBF mapping with G-TPS should only be used for rather coarse input meshes (number of vertices smaller than 1000). When RBF data mapping becomes too expensive, nearest-projection mapping is a good alternative except for very large out-of-memory cases. Scalability results for the mapping computation were recently published in 21. In future work, we aim for a more in-depth analysis of mapping variants with further geometries.

### 3.3 Communication

**Figure 12.**
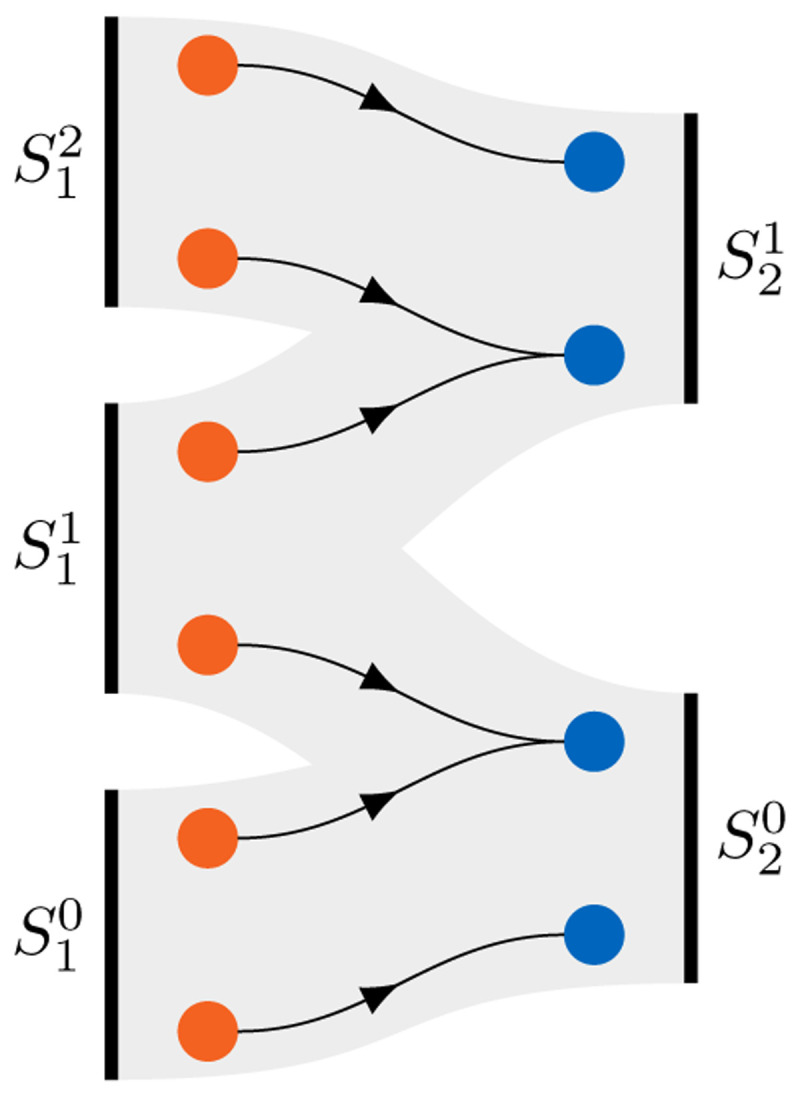
Communication initialization in preCICE. Given the distribution of vertices among the ranks of parallel participants $S_{1}^{i}$ and $S_{2}^{j}$ , combined with a data mapping between the vertices shown in the middle, preCICE deduces the required communication pattern of ranks between participants *S*_1_ and *S*_2_, depicted as the gray connections.

Besides coupling schemes and data mapping, the third feature pillar of preCICE is inter-code communication. For large-scale simulations on massively-parallel high-performance computing systems, efficient inter-code communication is a necessity. Employing any central instance not only deteriorates the communication performance, but can also be memory prohibitive when large amounts of data must be communicated. Therefore, preCICE implements fully-parallel point-to-point communication ^15, 17, 57^. In the initialization phase, preCICE performs an analysis of the coupling mesh partitions and the defined data mappings of each connected pair of participants to find the list of required connections between the MPI ranks of either participant (cf. Figure 12). To this end, bounding boxes around mesh partitions are compared in a first step, leading to preliminary communication channels. In a second step, actual mesh data is compared in a fully-parallel fashion ^21^.

As communication backends, preCICE supports MPI and TCP/IP. In general, communication via MPI is faster ^16^. However, TCP/IP communication is more robust and flexible, since not all MPI implementations support the necessary inter-code MPI functionality ^57^. For MPI-based communication, preCICE creates a single inter-code communicator including all involved ranks from both participants. To establish TCP/IP-based connections, on the other hand, each pair of connected ranks exchanges a connection token via the file system. To reduce the load on the file system, a hash-based scheme is used, which distributes the connection files uniformly across different directories ^6, 16^.

### 3.4 Getting and building preCICE

After introducing the basic coupling methods implemented in preCICE in the last three sections, we now give an overview of various ways to get preCICE. The GitHub repository ^7^ is the central platform for development, issue tracking, and contributing. It provides the release timeline with release notes, automatic source archives, and build artifacts. However, the repository only contains the preCICE library and native (C and Fortran) language bindings. Additional derived software is hosted in separate repositories under the preCICE GitHub organization: adapter codes, tutorials, Python and MATLAB language bindings, and more. All these components, together with the core library, are part of the *preCICE distribution* ^8^, a versioned and citable ecosystem of components that are meant to work together and are maintained by the preCICE developers. Everything presented in this paper refers to the version v2104.0 of the distribution ^19^.

On the other hand, the preCICE library also depends on various other libraries for a range of features. See Table 3 for an overview of dependencies and their associated features in preCICE.

**Table 3.** Dependencies of preCICE and associated features in preCICE.

Dependency	Version	CMake Option	Features
Boost Geometry	≥ 1.65.1	*required*	Spacial index trees
Boost Container	≥ 1.65.1	*required*	Flat maps and sets
Boost Stacktrace	≥ 1.65.1	*required*	Stacktrace information
Boost Log	≥ 1.65.1	*required*	Configurable logging
Boost Test	≥ 1.65.1	*required*	Base of testing framework
Eigen	3	*required*	Mesh representation and radial basis function mapping.
libxml	2	*required*	Parsing of XML config files.
PETSc	≥ 3.6	PRECICE_PETScMapping	Parallel RBF mapping.
Python	≥ 3.6	PRECICE_PythonActions	User-defined actions
NumPy	≥ 1.18.1	PRECICE_PythonActions	User-defined actions
MPI	MPI-3	PRECICE_MPICommunication	MPI communication back-end

**Figure 13.**
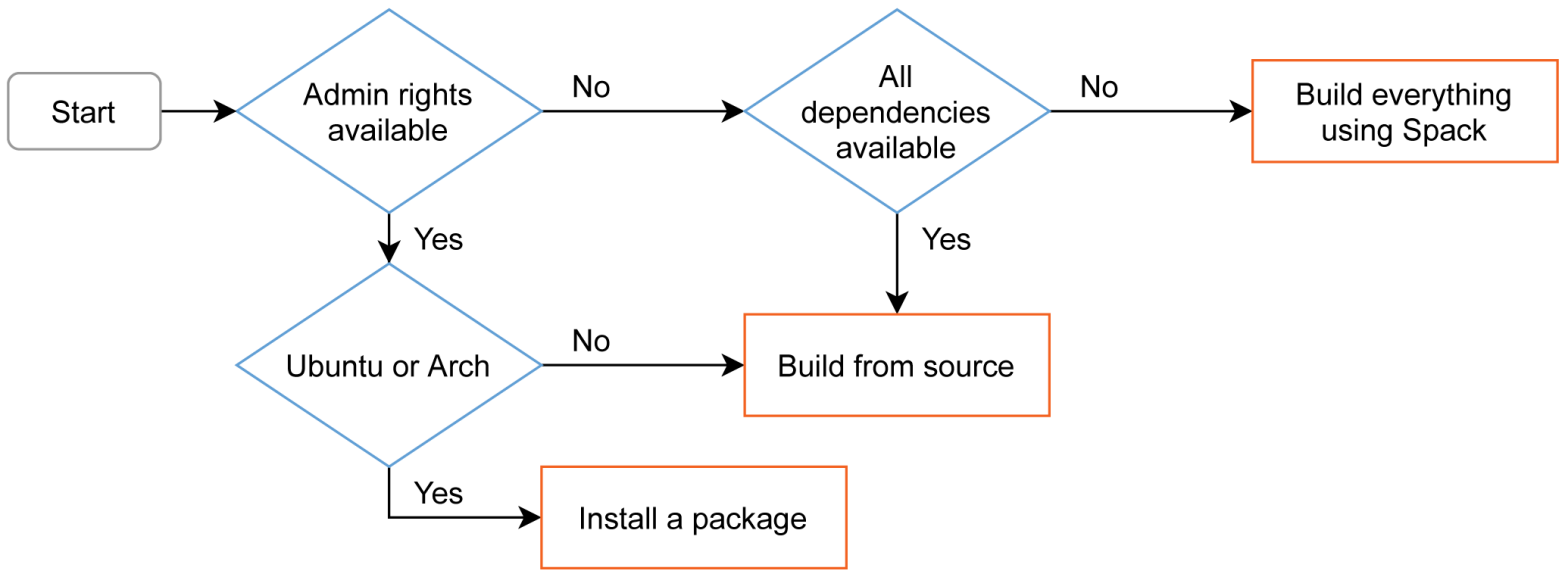
Decision graph and overview of the different installation methods for preCICE on Linux. On macOS, users can build using Spack, or install dependencies from Homebrew and build from source. On Windows, preCICE is available from MINGW (experimental), while it can also work inside the Windows Subsystem for Linux. Before deciding to install preCICE on their system, users can try a virtual machine containing all the software needed to run the preCICE tutorials.

A decision graph for getting preCICE is shown in Figure 13. We describe the different options in the following.

**Debian packages for preCICE on Ubuntu ** Due to the popularity of Ubuntu among preCICE users, we provide corresponding Debian packages. We aim to support the latest two Ubuntu *long term support* (LTS) releases, which is frequency-wise compatible with our strategy to not release new major versions (breaking changes) more often than every two to three years. The Debian packages contained in our GitHub releases allow one-click installation on supported platforms. This avoids explicit dependency management by the user. The current Debian package is always generated for the latest Ubuntu release as well as the latest Ubuntu LTS.

**Building preCICE using Spack** In addition, we maintain a Spack ^58^ package which allows to build the complete required software stack from source code. This is essential to be able to test arbitrary combinations of dependency versions and different compilers. We actively maintain a build recipe with common configurations. Moreover, preCICE is a member of the Extreme-scale Scientific Development Kit (xSDK) ^9^ since xsdk-0.5.0 (November 2019) ^10^, which promises compatibility to other major scientific computing packages.

**Building preCICE with CMake** For other platforms, we provide an in-depth guide on how to build preCICE from source. For cross-platform build system configuration, preCICE leverages CMake. It allows users and developers to develop and build in their environment of choice. The adoption of CMake simplifies package generation and the future support of Windows and macOS. macOS works out-of-the-box since preCICE v2.2. There are several ways to support Windows: Since v1.x, there is community support via MinGW. Windows users can also install preCICE on the WSL (Windows Subsystem for Linux) normally. We are currently preparing native Windows support (MSVC compiler), in addition.

**preCICE demo virtual machine** Before running their first coupled simulation, a user needs to install not only the preCICE library, but also a minimum set of adapters and third-party solvers. This can become even more complicated if the user does not already work on a platform compatible with all components. To lower the entry barrier, we provide a virtual machine (VM) image with all components needed to run the preCICE tutorials. We create ^11^ and distribute ^12^ this image as a Vagrant ^13^ Box, which is currently available for VirtualBox, but could easily be packaged for other providers as well. We chose Vagrant instead of a provider-specific system, as Vagrant allows us to highly automate the box generation, integrates with the host system automatically (SSH access, shared folders), provides infrastructure to distribute the box, and works with various host platforms and virtualization providers. We chose a VM instead of a container-based system, as virtual machines provide access to a full graphical environment by default and many users in our community already have experience working with virtual machines, but not with containers.

### 3.5 Application programming interface and configuration

Now that we know which coupling methods preCICE offers and how to get preCICE, we show in this section how preCICE can actually be used.

Even though preCICE is a C++ library, it also supports other programming languages, as shown in Table 4. Alongside the native application programming interface (API) for C++, preCICE provides C and Fortran bindings by default. The API for other languages is provided via independent projects, as they follow different release cycles, different project management, and different developer and installation procedures. Python bindings are based on Cython, installable via pip from PyPI. MATLAB bindings ^59^ are based on the MEX interface and Julia bindings are currently in the prototype phase. Finally, we develop an independent Fortran module for easier integration of preCICE into Fortran codes. The architecture and relation between these projects is described in the documentation ^14^. All language bindings also provide so-called *solverdummies* as example codes and provide pkg-config and CMakeConfig files for integration into other projects.

**Table 4.** Programming languages supported by preCICE. CMake options for C and Fortran bindings are set to ON by default.

Language	Location	Installation
C++	precice/precice	*native API*
C	precice/precice	cmake -DPRECICE_ENABLE_C=ON .
Fortran	precice/precice	cmake -DPRECICE_ENABLE_FORTRAN=ON .
Fortran Module	precice/fortran-module	make
Python	precice/python-bindings	pip install pyprecice@2.0.2
MATLAB	precice/matlab-bindings	MATLAB script
Julia	precice/julia-bindings	Julia package (experimental)

To introduce the API of preCICE, we use an example: we develop an adapter for a fluid solver written in Python to couple it to an already adapted solid solver for fluid-structure interaction (FSI). Mathematically, we realize a Dirichlet-Neumann coupling: we use the kinematic interface condition as Dirichlet boundary condition in the fluid solver and the dynamic interface condition as Neumann boundary condition in the solid solver. Thus, concerning coupling data, we receive the deformation of the solid from the solid solver as displacement values at the coupling interface and we return forces on the coupling interface to the solid solver. This example problem is representative for many preCICE users: an existing (in-house) fluid solver should be coupled to an off-the-shelf solid solver, which is already adapted for preCICE. The simplified code of the uncoupled fluid solver is depicted in Listing 1. u is the current solution, for example velocity and pressure values. We use an adaptive time step size, computed in line 3, and solve one time step in line 4.


1 u = initialize_solution()
2 while t < t_end: # main time loop
3      dt = compute_adaptive_dt()
4      u = solve_time_step(dt, u) # returns new solution
5      t = t + dt


**Listing 1.** Original uncoupled fluid solver in Python.

**Creating a handle to preCICE** Listing 2 shows the fully-coupled fluid solver. The preCICE API is used at multiple locations, which we explain in the following paragraphs. For the sake of simplicity, we do not develop a general stand-alone adapter, but directly use the preCICE API in the fluid code – meaning, we develop an *adapted code*. In Section 4, we give an overview of several *real* adapters. As the fluid code is written in Python, we make use of the Python bindings of preCICE: preCICE is imported in line 1. In line 3, the solver interface of preCICE is created. We pass the name of the solver and the preCICE configuration file. The latter defines the overall coupling topology (who is coupled to whom) and the used coupling methods (acceleration, data mapping, communication, etc.). We come back to this file later. Moreover, for parallel coupled codes, we need to give the current parallel rank (here 0) and the number of ranks (here 1) to preCICE. The solver interface is initialized in line 13. Here, preCICE performs several first steps, such as setting up internal data structures and creating communication channels. In the end, the solver interface is finalized in line 42. Internal data structures are torn down and communication channels are closed.


 1 import precice
 2
 3 interface = precice.Interface("Fluid", "precice-config.xml", 0, 1)
 4
 5 mesh_id = interface.get_mesh_id("Fluid-Mesh")
 6
 7 displ_id = interface.get_data_id("Displacement", mesh_id)
 8 force_id = interface.get_data_id("Force", mesh_id)
 9
10 positions = ... #define interface mesh, 2D array with shape (n, dim)
11 vertex_ids = interface.set_mesh_vertices(mesh_id, positions)
12
13 precice_dt = interface.initialize()
14
15 u = initialize_solution()
16
17 while interface.is_coupling_ongoing(): # main time loop
18
19      if interface.is_action_required(precice.action_write_iteration_checkpoint()):
20           u_checkpoint = u
21           interface.mark_action_fulfilled(precice.action_write_iteration_checkpoint())
22
23      # returns 2D array with shape (n, dim)
24      displacements = interface.read_block_vector_data(displ_id, vertex_ids)
25
26      dt = compute_adaptive_dt()
27      dt = min(precice_dt, dt)
28      u = solve_time_step(dt, u, displacements) # returns new solution
29
30      # returns 2D array with shape (n, dim)
31      forces = compute_forces(u)
32      interface.write_block_vector_data(force_id, vertex_ids, forces)
33
34      precice_dt = interface.advance(dt)
35
36      if interface.is_action_required(precice.action_read_iteration_checkpoint()):
37           u = u_checkpoint
38           interface.mark_action_fulfilled(precice.action_read_iteration_checkpoint())
39      else: # continue to next time step
40           t = t + dt
41
42 interface.finalize()



**Listing 2.** An adapted fluid solver written in Python. While preCICE is a C++ library, bindings for C++, C, Fortran, Python, and MATLAB make it possible to couple a large variety of participants in a minimally invasive way.

**Coupling meshes ** Coupling data and coupling meshes are referred to by IDs, which are collected in lines five to eight. The coupling mesh is defined before the initialization in line 11. preCICE treats coupling meshes as (unstructured) clouds of vertices, arranged in two-dimensional arrays of size vertices by dimension. Certain features of preCICE (e.g., nearest-projection data mapping) require mesh connectivity, in addition. To this end, edges, triangles, and quads can optionally be defined, a step which we do not show in this example. The control of the end of the simulation is handed over to preCICE in line 17 to steer a synchronized end of all participants. On the coupling mesh, coupling data structures are accessed in lines 24 and 32. The example uses specific calls for the vector-valued displacement and force values. The displacement values are used as Dirichlet boundary condition, here depicted as additional input of solve_time_step in line 28. The force values are computed from the current solution by means of a helper function in line 31.

**Figure 14.**
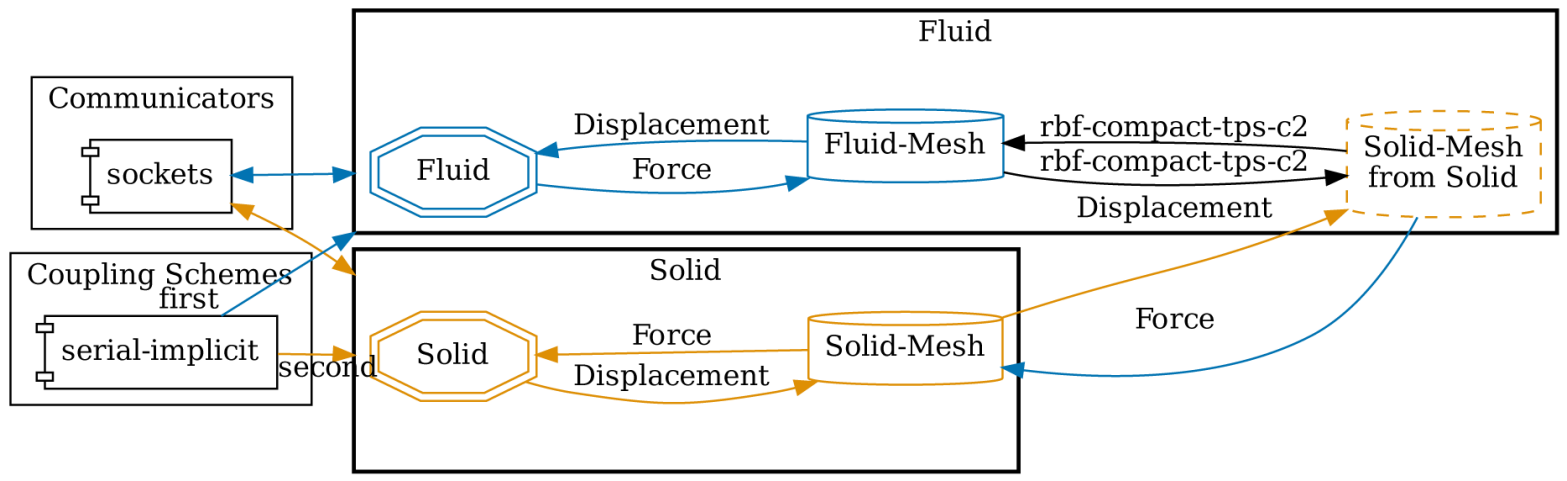
Auto-generated visualization of the preCICE configuration of Listing 3 using the *Config Visualizer* of preCICE.

**Configuration** Listing 3 gives an excerpt of a preCICE configuration for our FSI example. The dimension of the scenario is specified in line 1 and can be either 2 or 3. Two participants, Fluid and Solid, are configured. Fluid uses the mesh from Solid in line 13. This way, we can define data mappings between both meshes in lines 16 and 17. Here, we use RBF data mappings with compact thin-plate splines as basis functions. In line 22, we configure a TCP/IP sockets connection between both participants. Finally, in lines 24 to 31, a serial implicit coupling between both participants with an IQN-ILS acceleration is defined. Fluid is the first participant, meaning that it starts each iteration. preCICE comes with a standalone Python tool called *Config Visualizer* ^15^, which helps understanding and debugging preCICE configuration files. The tool generates graphviz dot files ^60^, which can be, for example, converted to PDF. The generated PDF output is shown in Figure 14 for the example configuration.


 1 <solver-interface dimensions="3">
 2   <data:vector name="Force"/>
 3   <data:vector name="Displacement"/>
 4
 5   <mesh name="Fluid-Mesh">
 6     <use-data name="Displacement"/>
 7     <use-data name="Force"/>
 8   </mesh>
 9   <mesh name="Solid-Mesh"> ... </mesh>
10
11   <participant name="Fluid">
12     <use-mesh name="Fluid-Mesh" provide="yes"/>
13     <use-mesh name="Solid-Mesh" from="Solid"/>
14     <write-data name="Force" mesh="Fluid-Mesh"/>
15     <read-data name="Displacement" mesh="Fluid-Mesh"/>
16     <mapping:rbf-compact-tps-c2 from="Fluid-Mesh" constraint="conservative"/>
17     <mapping:rbf-compact-tps-c2 from="Solid-Mesh" constraint="consistent"/>
18   </participant>
19
20   <participant name="Solid"> ... </participant>
21
22   <m2n:sockets from="Fluid" to="Solid" />
23
24   <coupling-scheme:serial-implicit>
25     <participants first="Fluid" second="Solid"/>
26     <time-window-size value="1e-3"/>
27     <exchange data="Force" mesh="Solid-Mesh" from="Fluid"/>
28     <exchange data="Displacement" mesh="Solid-Mesh" from="Solid"/>
29     ...
30     <acceleration:IQN-ILS> ... </acceleration:IQN-ILS>
31   </coupling-scheme:serial-implicit>
32 </solver-interface>



**Listing 3.** Excerpt of a preCICE configuration file. Two participants Fluid and Solid are coupled.

**Figure 15.**
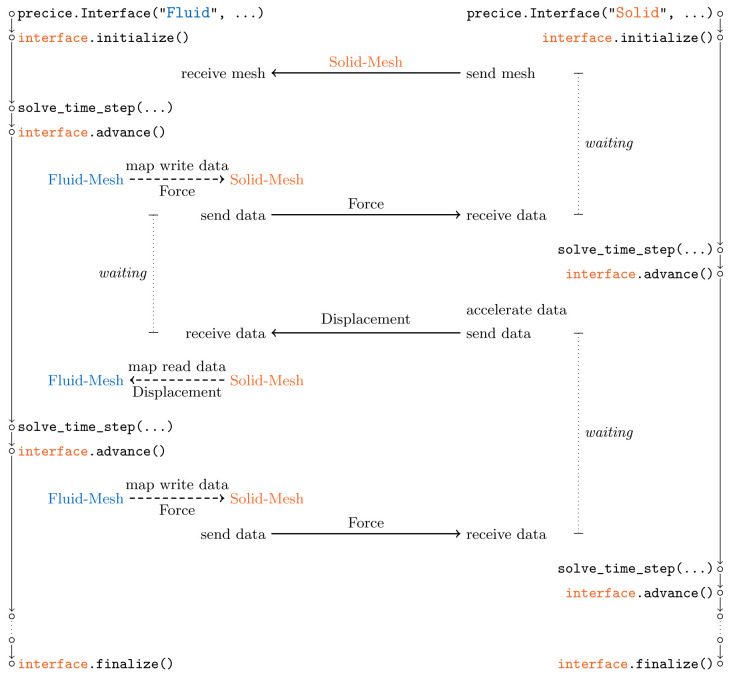
Overall flow of coupling steps resulting from the preCICE configuration of Listing 3. The serial coupling scheme leads to a staggered execution of both participants, one after the other. Both participants wait in initialize and advance for synchronization. We assume identical time step sizes in both participants.

**Coupling flow** The actual coupling between participants happens within the single preCICE API function advance, line 34 in Listing 2. This includes communication, mapping, and acceleration of coupling data – whatever methods are defined in the preCICE configuration. To better understand the order and relation of individual coupling steps, Figure 15 depicts the overall coupling flow when using the example configuration of Listing 3. For this visualization, we further assume that both participants use identical time step sizes. During the initialization, Solid-Mesh is sent from Solid to Fluid. A serial coupling scheme leads to a staggered execution of both participants: one after the other. This implies, in particular, that the behavior of both participants within the preCICE API functions cannot be symmetric. In the example, Fluid is the first participant of the coupling scheme. This means that, after the first time step of Fluid, the first advance sends force values to Solid, as can be seen in the figure. This coupling data is, however, already received in initialize of Solid, such that the solver can use it in its first time step. The first displacement values are then sent at the start of the first advance of Solid and received at the end of the first advance of Fluid. Data is mapped in both direction within advance of Fluid. Convergence acceleration of the coupling iteration is always executed in advance of the second participant, here Solid. Please note that a different preCICE configuration could lead to a completely different order and relation of steps: the roles of first and second could be swapped, one or both data mappings could be computed on Solid, or the serial coupling scheme could be replaced by a parallel one, to only name a few choices. All these changes can be configured at runtime. The adapted fluid code in Listing 2 remains unchanged – and would remain unchanged even if Solid would be coupled with a third participant.

**Timestepping** So far, we assumed that the coupled participants use matching time step sizes. preCICE is, however, also able to handle non-matching time step sizes. Then, data is only exchanged at the end of each time window, defined in the preCICE configuration, line 26 of Listing 3. Alternatively, the time window size can also be imposed by the first participant. If a solver uses a smaller time step size than the time window size, it subcycles within the time window. This means, in particular, that the same coupling data is used throughout the time window, which can reduce the time discretization order of the coupled codes. We are currently working on a higher order time representation of coupling data to sample from, a coupling procedure known as waveform iteration ^40^. To allow preCICE to track the time of a solver, the current time step size needs to be passed to preCICE in advance, cf. line 34 in Listing 2. preCICE then returns the remaining time within the current time window, which the coupled solver has to respect. Therefore, in line 27, the solver’s time step size is restricted, if required.

**Implicit coupling** We still need to explain how implicit coupling is realized. Please remember that by implicit coupling we mean the repetition of time windows until sufficient convergence of coupling data (cf. Section 3.1). To this end, a coupled solver needs to be able to move backwards in time, which we realize by writing and reading checkpoints of the complete internal solver state (lines 20 and 37 in Listing 2). Writing checkpoints is required when entering time windows for the first time and reading checkpoints is required at the end of a time window, whenever convergence is not achieved. As the solver does not know anything about the coupling scheme, preCICE tells the solver when it is time to write and read checkpoints in lines 19 and 36. At the end of the loop body, time is only increased when convergence is achieved, in line 40. Please note that, with this checkpointing mechanism, nested time and coupling loops are not necessary, but everything can be handled within one while loop.

**Help and further information** We cannot explain all preCICE API functions and all configuration options with this single example. Please also consider the official user documentation ^16^, which includes complete API and configuration references. preCICE supports adapter development by extensive sanity checks of correct API usage – a simple example: advance cannot be called before initialize. Moreover, the preCICE configuration is checked against the configuration reference and extensive logging is configurable.

## 4 Official adapters

A library such as preCICE can only live as part of an application (a solver) that calls it. To call preCICE, the solver needs to contain code that knows how to interact with the library. We saw in Section 3 that this additional code is short, but the user should be able to start setting up a coupled simulation at the level of describing a scenario, not at the level of writing code for each of the involved solvers.

To lower the entry barrier and to make sure that the majority of users can keep using popular solvers with the latest versions of preCICE, we have developed a set of official adapters, which we host and maintain in their own repositories under the preCICE GitHub organization ^17^. This allows each project to follow a fitting development cycle and makes it easier for the community to contribute and to adopt projects upstream. As the collection of adapters grows, such community contributions are crucial, not only in fixes and features, but also in assuming maintainer roles.

We present here all mature official adapters to date. All the solvers discussed in this section are free/open-source projects, a fundamental property that greatly facilitates the adapter development and distribution. For free/open-source solvers, adapters can have the form of (i) in-place source code modifications, (ii) calls to an additional adapter class, or (iii) runtime plugins, wherever supported. In contrast to adapters for open-source solvers, coupling of closed-source solvers usually entails interacting through a wrapper, application programming interface (API), or control files, architectures which potentially cancel fundamental features of preCICE.

We begin with OpenFOAM and SU2, two solvers primarily used for simulating fluids. We continue with CalculiX and code_aster, two solvers primarily used for simulating solids. We then discuss FEniCS, deal.II, and Nutils, general FEM frameworks for which we provide various coupled examples. At the end of the section, we list further adapters maintained by the community.

### 4.1 OpenFOAM

OpenFOAM ^18^ is a finite volume toolbox and collection of solvers primarily for CFD simulations ^61^. The OpenFOAM adapter ^19^ is currently the most frequently used of the listed adapters and OpenFOAM represents the fluid solver in most of our tutorial cases. It is also the adapter with the highest number of contributions in the context of student and research projects ^62– 65^. The adapter is being actively developed and more features have been added in the past years by multiple contributors. A separate reference publication for the OpenFOAM-preCICE adapter is currently under review by the OpenFOAM ^20^.

On the technical side, the adapter is an OpenFOAM function object, to which OpenFOAM can link at runtime. Function objects are *plug-ins* that OpenFOAM uses mainly for optional post-processing tools. Implementing the adapter in this way allows using the adapter with any standard or in-house OpenFOAM solver (each being a stand-alone application) that supports function objects, without modifying the code of the solver ^63^. The separation between the solver and the adapter has facilitated development and increased user adoption, such that we now aim for this model wherever possible. We support the latest versions of the major OpenFOAM variants, including v1706–v2106 (ESI/OpenCFD, main adapter branch) and 4.0–8 (The OpenFOAM Foundation, version-specific branches). The adapter can be built from source using the WMake build system of OpenFOAM and installed into the FOAM_USER_LIBBIN directory.

On the application side, the adapter supports conjugate heat transfer (CHT), fluid-structure interaction (FSI), and fluid-fluid coupling. In terms of CHT, it can read and write temperature, heat flux, sink temperature, and heat transfer coefficient, allowing not only for Dirichlet-Neumann, but also for Robin-Robin coupling. 

In terms of FSI, the adapter can read absolute and relative displacements (defined on either face nodes or face centers), while it can write forces and stresses (on face centers). At least the mesh motion solver displacementLaplacian is known to work. Again, a distinction between compressible and incompressible solvers is needed.

The adapter also supports fluid-fluid coupling, reading and writing pressure, velocity, as well as their gradients. This is an area of active research and further development.

The coupling fields, patch names, participant name, path to the preCICE configuration file, and more are configured in the adapter configuration file system/preciceDict, an OpenFOAM dictionary.

In addition to that, the user needs to specify the adapter function object in the system/controlDict. Several tutorial cases are available, using the solvers pimpleFoam, buoyantPimpleFoam, buoyantSimpleFoam, and laplacianFoam. There are also several examples in which the preCICE community has used the adapter (as-is or modified) with further standard and in-house OpenFOAM solvers, including cases with compressible multiphase flow ^66^ and cases with volume coupling ^67– 69^.

The code is available on GitHub ^21^ under the GPLv3 license, the same license as OpenFOAM. The code contains also comments with instructions on extending it.

The OpenFOAM community is currently developing (and has already done so in the past) very important contributions in bringing multi-physics simulations to OpenFOAM. Prominent examples include the standard CHT solver chtMultiRegionFoam ^22^ and the FSI solvers fsiFoam ^70^ and solids4Foam ^71^. These projects solve the respective multi-physics problem monolithically, at least software-wise: they implement both single-physics domains inside OpenFOAM, compiled in the same executable. In contrast, the OpenFOAM-preCICE adapter provides additional flexibility to couple OpenFOAM with any other solver via preCICE. Other projects also apply the partitioned approach to extend OpenFOAM with the functionality of other codes, including OpenFPCI (ParaFEM) ^72^, EOF-Library (Elmer) ^73^, and ATHLET-OpenFOAM coupling ^74^. OpenFOAM has previously also been coupled with preCICE using independent (unofficial) adapters in the theses of Kevin Rave ^75^ (CHT) and David Schneider ^76^ (FSI), as well as in the project FOAM-FSI of David Blom for foam-extend ^23^. The official OpenFOAM-preCICE adapter differs in providing a general-purpose adapter for preCICE for a wide range of users and use cases.

### 4.2 SU2

SU2 ^24^ (Stanford University Unstructured) is a finite volume solver which provides compressible and incompressible solver variants for CFD ^77^. The SU2 adapter ^78^ supports SU2 v6.0 “Falcon” and contributions from the community are particularly welcome in this project ^25^.

As SU2 is written in C++, the adapter directly uses the C++ API of preCICE. The API calls are provided by an adapter class, which is utilized in the SU2 solver files (e.g., in SU2_CFD.cpp). An installation script copies the modified, version-specific files to specific locations in the SU2 source code, which is then built normally.

The adapter is designed for FSI applications and supports reading forces and writing absolute or relative displacements. The adapter is configured via additional options in the native configuration file of SU2 and the modified solver can be executed with or without enabling preCICE. The user can set the name of the marker which identifies the FSI interface in the geometry file of SU2 and can run simulations with multiple coupling interfaces.

Similarly to the adapter, SU2 is also used as a solver in other coupling projects, for example CUPyDO ^79^, where the CUPyDO coupler calls SU2 via a Python wrapper. In addition to external coupling options, SU2 offers monolithic capabilities for multi-physics simulation such as FSI and CHT ^80, 81^.

### 4.3 CalculiX

CalculiX ^26^ is an open-source FEM code ^82^. CalculiX offers a variety of solvers and the CalculiX adapter ^27^ enables coupling some of these solvers via preCICE. The adapter supports the *dynamic linear geometric* and the *dynamic nonlinear geometric* solvers of CalculiX for coupled FSI problems, as well as *static thermal* and *dynamic thermal* solvers for CHT problems. The CalculiX adapter ^20, 62^ is compatible with CalculiX 2.16, it is regularly updated for new CalculiX releases, and maintains support for older versions in version-specific branches.

The adapter directly modifies the source code of CalculiX and produces a stand-alone executable ccx_preCICE, which can be used both for coupled and for CalculiX-only simulations: the flag -precice-participant <name> enables the preCICE adapter. All preCICE-related functionality is provided in additional source files supplied with the adapter.

The adapter is configured through a YAML file that specifies the coupling interface names, coupling data variable types, and type of interface (mesh nodes, or mesh nodes with connectivity). It can be used with both linear and quadratic tetrahedral (C3D4 and C3D10) and hexahedral (C3D8 and C3D20) solid elements, as well as S3 and S6 tetrahedral shell elements.

CalculiX is written in C and Fortran. However, all preCICE functionality is incorporated using the C bindings of preCICE. To perform coupled simulations with CalculiX in parallel on shared-memory systems, the adapter treats CalculiX as a serial participant, while the CalculiX linear solver is executed in parallel. More details on how to run CalculiX in parallel is available in the CalculiX user manual ^83^.

### 4.4 code aster

code_aster ^28^ is an FEM code in Fortran (with a Python API) developed by EDF France, offering solvers for heat transfer, structural analysis, and more, with one of the main applications being nuclear power engineering. The code_aster adapter ^62^ ^29^ is compatible code_aster 14.6, while it is being maintained to work with the latest versions of preCICE.

On the technical side, the adapter is a single adapter.py file providing methods that are used in an example adapter.comm command file for CHT simulations. As a Python code, the adapter depends on the preCICE Python bindings. It can be installed by copying the adapter file into the ASTER_ROOT/14.6/lib/aster/Execution/ directory.

On the application side, the adapter can currently read and write sink temperature and heat transfer coefficient, thus supporting Robin-Robin coupling for CHT. The preCICE tutorials include such a Robin-Robin CHT case with code_aster and the steady-state fluid solver buoyantSimpleFoam.

The code is available on GitHub ^30^ under the GPLv2 license and can be easily extended by adding more coupling fields in adapter.py and providing their names as arguments to the method adapter.writeCouplingData().

### 4.5 FEniCS

FEniCS is an open-source general-purpose FEM package with a high-level Python interface ^84^. FEniCS does not provide ready-to-use solvers, but instead provides a broad range of tools for solving partial differential equations with a high level of abstraction. A wide variety of examples is provided in the FEniCS project to illustrate its usage ^85^. The FEniCS-preCICE adapter ^86^ facilitates coupling of FEniCS-based solvers using preCICE.

The adapter is configured using a JavaScript object notation (JSON) file. Afterwards, the adapter is initialized by providing a FEniCS Mesh and a SubDomain to define the coupling boundary.

For data exchange, the adapter offers a simple function adapter.write_data(solution). This function samples a given solution on the previously defined coupling mesh and writes the samples to preCICE. The function coupling_data = adapter.read_data() returns the interface data provided by preCICE. The adapter provides two possibilities to transform this raw coupling_data to a boundary condition that can be used in FEM: (1) An Expression can be generated and used as a functional representation of provided coupling_data via interpolation or (2) a PointSource can be generated to apply point-wise loads. Both approaches have their respective use-cases for FEniCS users ^86^, but a user can also use the raw coupling_data to create boundary conditions depending on the individual requirements.

The adapter supports the built-in parallelism of FEniCS and uses its domain decomposition. If the coupling interfaces are decomposed over multiple ranks, the adapter implements additional inter-process communication at the interface between two ranks.

Many packages similar to FEniCS exist, such as firedrake ^87^, or the FEniCS successor FEniCS-X ^31^. The adapter is not designed to work with these packages, but it can serve as a template for the development of specialized adapters.

The adapter is distributed under the LGPLv3.0 license on PyPI ^32^. If preCICE is installed on the system, the latest version of the adapter can be installed via pip. With FEniCS being a Python-based package, the adapter depends on the preCICE Python bindings, which are automatically installed with the adapter. The source code of the adapter is available on GitHub ^33^ and user documentation can be found on the preCICE website ^34^.

Related work to solve multi-physics problems with FEniCS includes, for example, the monolithic fluid-structure interaction solver turtleFSI ^88^ written in FEniCS, as well as FENICS-HPC ^89^. FEniCS extensions such as multiphenics ^35^ have also been developed to promote prototyping of multi-physics problems.

### 4.6 deal.II

deal.II ^90, 91^ is a general-purpose FEM library written in C++. Similar to FEniCS, deal.II does not include ready-to-use solvers, but allows users to write their own application codes by providing an easy-to-use interface to complex FEM-specific data structures and algorithms. The library provides state-of-the art numerical techniques and their implementations leverage distributed memory computations, vectorization, threading and matrix-free implementations, which have been proven to scale up to whole supercomputers ^92, 93^.

While deal.II is a general-purpose library, the deal.II adapter focuses on a subset of relevant applications and features. Instead of trying to provide a general-purpose deal.II adapter, we provide examples for users that want to develop their own preCICE-enabled solvers with deal.II. These examples ^36^ show linear and non-linear elastic solid mechanics codes in a coupled FSI scenario. From a user perspective, these coupled codes are ready-to-use without detailed knowledge of deal.II itself, but can also provide a starting point for own application-specific adapter developments. Similarly to deal.II and preCICE, the examples are built using CMake and a parameter file controls solver-specific preCICE settings. Simple meshes can directly be defined in the source code, whereas external meshes can be loaded at runtime.

In addition to these examples, we have created a very basic stand-alone one-way coupling example and contributed it to the deal.II code-gallery ^37^. In this example, a Laplace problem is coupled to a time-dependent C++ boundary condition code. The example is meant to serve as a first impression of how the preCICE API looks and how to use it along with deal.II.

As the low-level design of deal.II offers a lot of freedom of implementation approaches, multi-physics simulations have also been implemented in other ways ^94, 95^.

### 4.7 Nutils

Similar to FEniCS and deal.II, Nutils ^96^ is also a general-purpose FEM library. Missing capabilities for distributed computing and the fact that Nutils is purely written in Python, including matrix assembly, makes the library somehow less performant than alternatives. The powerful and intuitive API of Nutils, however, allows for fast prototyping. These points make Nutils a perfect option for the *cheaper*, but possibly more complex participant of a coupled simulation, or for testing new coupling approaches. A first partitioned heat conduction example coupling two Nutils participants was developed and validated within only a half day of work and is available as a preCICE tutorial (cf. Section 5). In general, coupling a new Nutils application code is a rather simple task and can be realized best by copying and adapting existing examples. Defining coupling meshes and accessing coupling data is a particularly simple task as illustrated in Listing 4. Therefore, in contrast to (for example) FEniCS, developing a general stand-alone Nutils-preCICE adapter is not necessary. In recent years, several examples have been realized. The preCICE documentation gives an up-to-date overview ^38^.


 1 import precice, nutils
 2
 3 domain = ... # define Nutils domain
 4 ns.u = ... # Nutils solution u in namespace ns
 5 interface = precice.Interface("FluidSolver", "precice-config.xml", 0, 1)
 6 [...]
 7 # defining a coupling mesh
 8 coupling_boundary = domain.boundary['top']
 9 coupling_sample = coupling_boundary.sample('gauss', degree=2)
10 vertices = coupling_sample.eval(ns.x)
11 vertex_ids = interface.set_mesh_vertices(meshID, vertices)
12 
13 # instead of Gauss points, we can also couple at (sub-sampled) cell vertices
14 coupling_sample = couplinginterface.sample('uniform', 4) # 4 sub samples per cell
15
16 # or volume coupling
17 coupling_sample = domain.sample('gauss', degree=2)
18
19 # reading coupling data and applying as boundary condition
20 read_data = interface.read_block_scalar_data(read_data_id, vertex_ids)
21 read_function = coupling_sample.asfunction(read_data)
22 sqr = coupling_sample.integral((ns.u - read_function)**2)
23 constraints = nutils.solver.optimize(sqr, ...) # for a Dirichlet BC
24
25 # writing data
26 write_data = coupling_sample.eval('u' @ ns, ...)
27 interface.write_block_scalar_data(write_data_id, vertex_ids, write_data)


**Listing 4.** A simplified coupled Nutils code. Due to the rich and flexible API of Nutils, defining coupling meshes and accessing coupling data are simple tasks, rendering a stand-alone Nutils-preCICE adapter unnecessary.

### 4.8 Further adapters

The aforementioned are not the only adapters published in the preCICE GitHub organization. The organization also includes a few less-actively maintained projects, which mainly serve as starting points for anyone who wants to build a more complete solution. If this applies to you, we would appreciate your feedback and contributions, especially in tutorial cases and maintenance.

- ANSYS Fluent: Intended for the fluid part in FSI and implemented as a so-called *user-defined function* plug-in. This adapter ^14, 97^ is currently experimental ^39^.
- COMSOL Multiphysics: Intended for the structure part in FSI. Similarly to Fluent, this is one of the earliest adapters and it is currently not actively maintained.
- MBDyn: Intended for the structure part in FSI. Contributed by the TU Delft Wind Energy group and irregularly extended by the community ^23^. The adapter repository ^40^ includes a tutorial case which simulates 3D cavity flow with a flexible bottom surface in which MBDyn is coupled to OpenFOAM.
- LS-DYNA: Intended for the structure part of CHT. Not a ready-to-use adapter, but rather a detailed description on how to create an actual LS-DYNA adapter. Contributed by the LKR group at the Austrian Institute of Technology ^69^.
- Elmer FEM: Intended for the structure part in FSI. Currently under development in a student project ^41^.

Apart from these codes, you can also find a list of community-developed projects in Section 7.

## 5 Illustrative examples

After installing preCICE, a user typically wants to run a first coupled example case as close to their application and preferred solvers as possible. Such an example needs to be simple enough to follow without significant expertise in any of the involved solvers, but yet full-featured in terms of coupling. With this in mind, we offer a collection of tutorial cases hosted on precice/tutorials, with step-by-step guides in the preCICE documentation ^42^. Such a tutorial consists of all the required instructions and configuration files necessary to run the coupled simulation, as well as convenience scripts to run, visualize, and cleanup each case. The same cases and scripts are also used in the preCICE system tests (cf. Section 6.3) and all tutorials follow a consistent structure and naming scheme, described in the contributing guidelines ^43^.

An important design decision of these tutorials is that every combination of the available solvers should work and give reasonably similar results, demonstrating the plug-and-play concept of preCICE. This lets the user start from a case as close to their target as possible and then potentially replace one of the participants with their own solver, maintaining a reference to compare with. To achieve this goal, we had to develop features that may otherwise seem unnatural. One of the most prominent such features is that the adapters for OpenFOAM and CalculiX (natively 3D solvers) can also work in a *2D mode*, coupling only lines of face points instead of surfaces. This automatic 2D mode works either by applying additional interpolation to map points from the mesh nodes to the face centers, or directly switching to data available at the face centers or to data on a predefined plane. 

**Figure 16.**
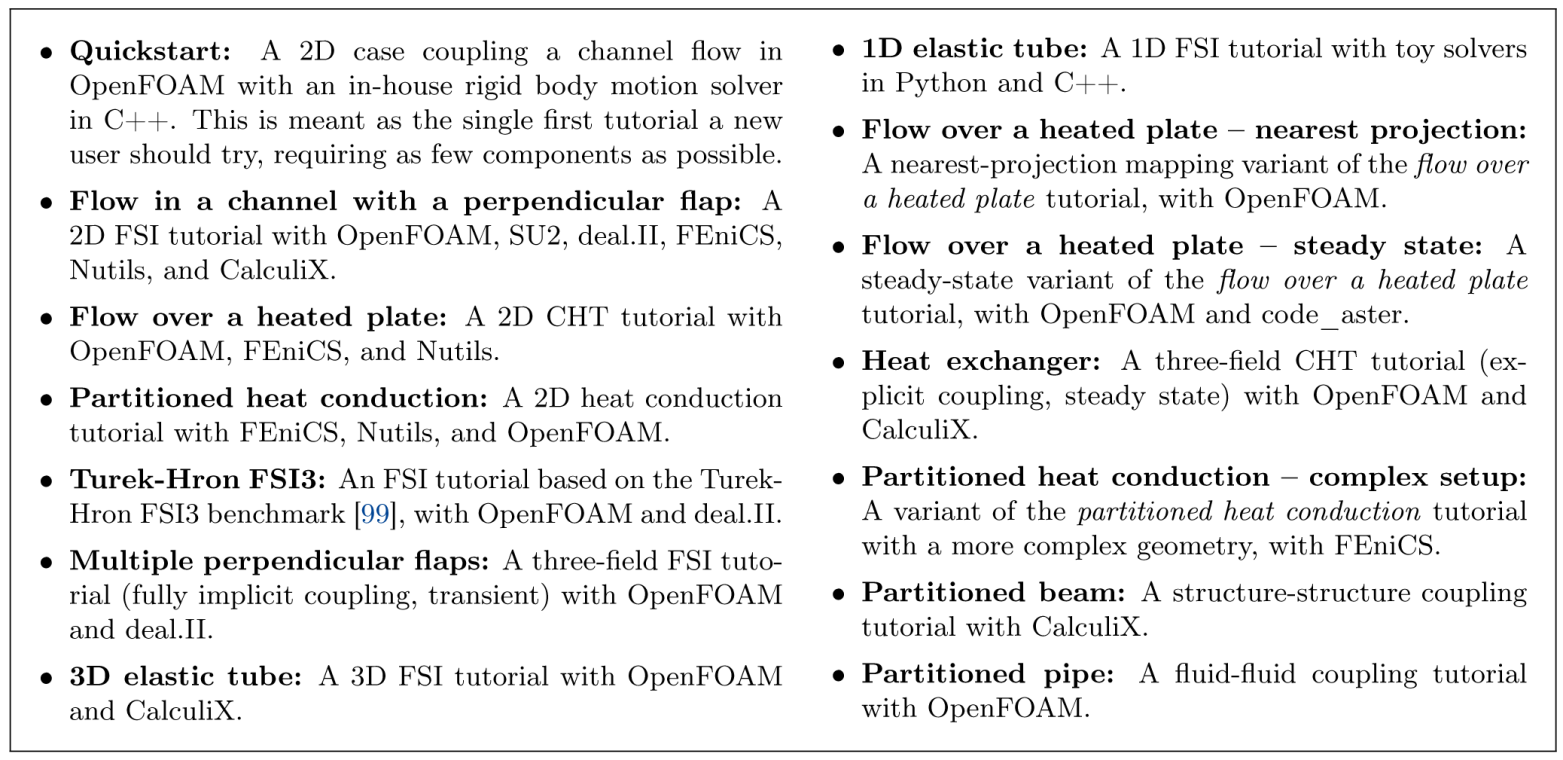
List of available tutorial cases.

Historically, the collection has grown with contributions from the community. As such, the tutorials should currently be seen as individual examples showcasing particular applications and features, rather than as a structured cookbook. We focus here on two representative cases that are available for a wide range of solvers: a CHT and an FSI example. The complete collection of tutorials is listed in Figure 16.

### 5.1 Flow over a heated plate CHT tutorial

**Figure 17.**
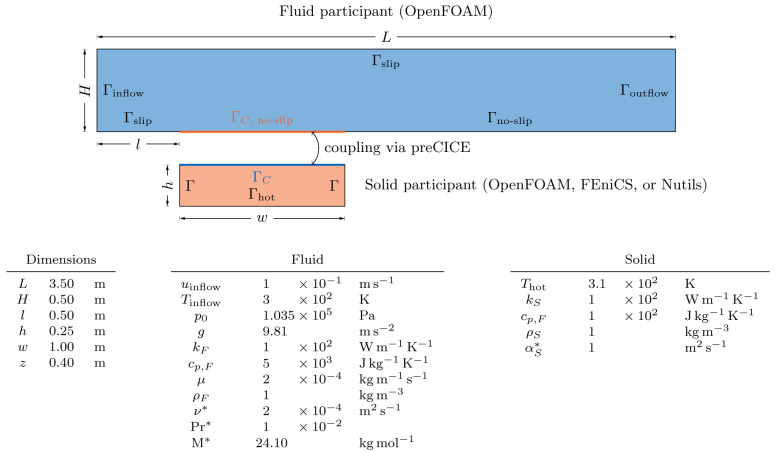
Flow over a heated plate CHT tutorial: The setup is depicted at the top, geometric and physical parameters are listed in the tables at the bottom of the figure. The fluid participant reads heat flux at the interface Γ *_C_*, while the solid participant reads temperature. The boundary values for the inflow (Γ _inflow_), outflow (Γ _outflow_), and the hot bottom of the plate (Γ _hot_) are listed in the tables at the bottom of the figure. All other boundaries are insulated. *u*_inflow_, *T*_inflow_: velocity and temperature at the inflow boundary. *p*_0_: ambient pressure at all boundaries of the fluid. *T*_hot_: temperature at the bottom of the plate. *g*: acceleration due to gravity. *k*_F_ and *k*_S_: thermal conductivity of the fluid and solid. *ρ _F_* and *ρ _S_*: density of the fluid and solid. *c _p,F_* and *c _p,S_*: specific heat capacity of the fluid and solid. $\alpha_{S}^{*}$ = *k _S_* /( *ρ _S_c _p,S_*): thermal diffusivity of the solid. *µ*: dynamic viscosity. ν* = *µ*/ *ρ _F_*: kinematic viscosity. Pr* = *c _p,F_ µ*/ *k _F_*: Prandtl number of the fluid. M* = *_ρF_* *RT*_inflow_/ _*p*_0__: Molar mass of the fluid with *R* being the gas constant. *z* is the out-of-plane thickness: even if the coupled case is described as 2D, OpenFOAM is still a 3D solver. Quantities marked with a * are derived quantities.

**Figure 18.**
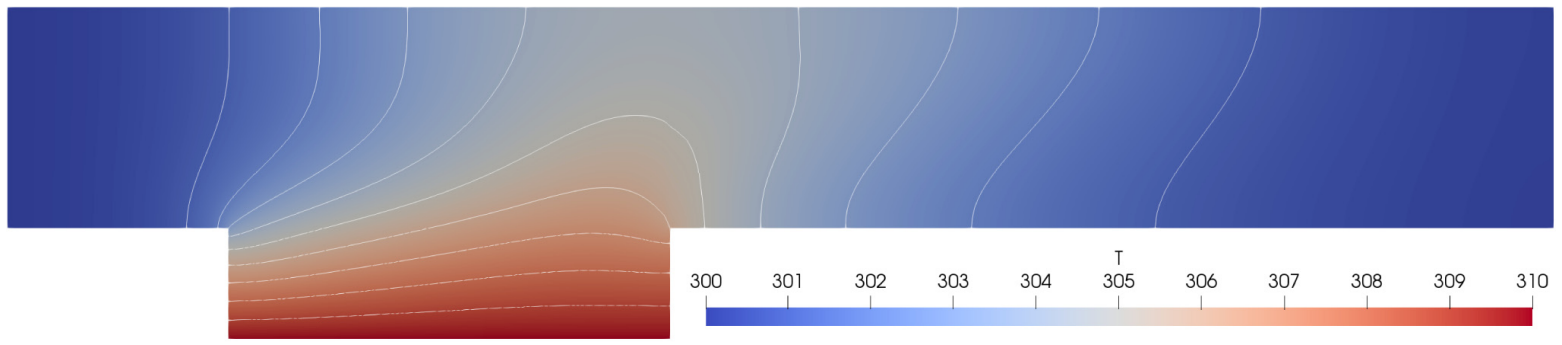
Flow over a heated plate CHT tutorial: Isothermal lines for the OpenFOAM-FEniCS combination at time *t* = 10 *s*. The lines are continuous and smooth across the interface. Similar results are observed for all other solver combinations.

This tutorial consists of a simple CHT scenario. The case consists of a 2D channel flow, coupled at its bottom with a 2D heated solid plate (cf. Figure 17). As heat is conducted across the solid plate, the temperature of the flow region above and downstream the plate increases, as shown in Figure 18. We discuss here the transient variant of this tutorial ^44^. Since experimental data is available by Vynnycky *et al.* ^98^ the scenario serves often as a validation case for CHT simulations ^99, 100^.

The fluid participant is the compressible OpenFOAM solver buoyantPimpleFoam. For the solid participant, the user can choose among the OpenFOAM solver laplacianFoam and heat conduction solver examples based on FEniCS or Nutils. In the case of laplacianFoam, we compute the heat flux assuming a constant heat conductivity *k _S_*, which is additionally specified in the OpenFOAM adapter. In the case of FEniCS, the solver was developed ^40^ based on a heat equation example from Langtangen *et al*. ^85^. A detailed description of the solver can be found in the FEniCS adapter reference paper ^86^. In case of Nutils, we provide a similar example.

All of the possible combinations ({OpenFOAM} × {OpenFOAM, FEniCS, Nutils}) use the same preCICE configuration file and the user can select any combination at runtime. The solid participant solvers write heat flux values and apply a Dirichlet boundary condition by reading temperature values at the coupling interface. Accordingly, the fluid OpenFOAM participant writes temperature values and applies a Neumann boundary condition by reading heat flux values at the coupling interface. By default, the tutorial is configured with a serial-implicit coupling scheme in combination with Aitken under-relaxation and nearest-neighbor mappings.

**Figure 19.**
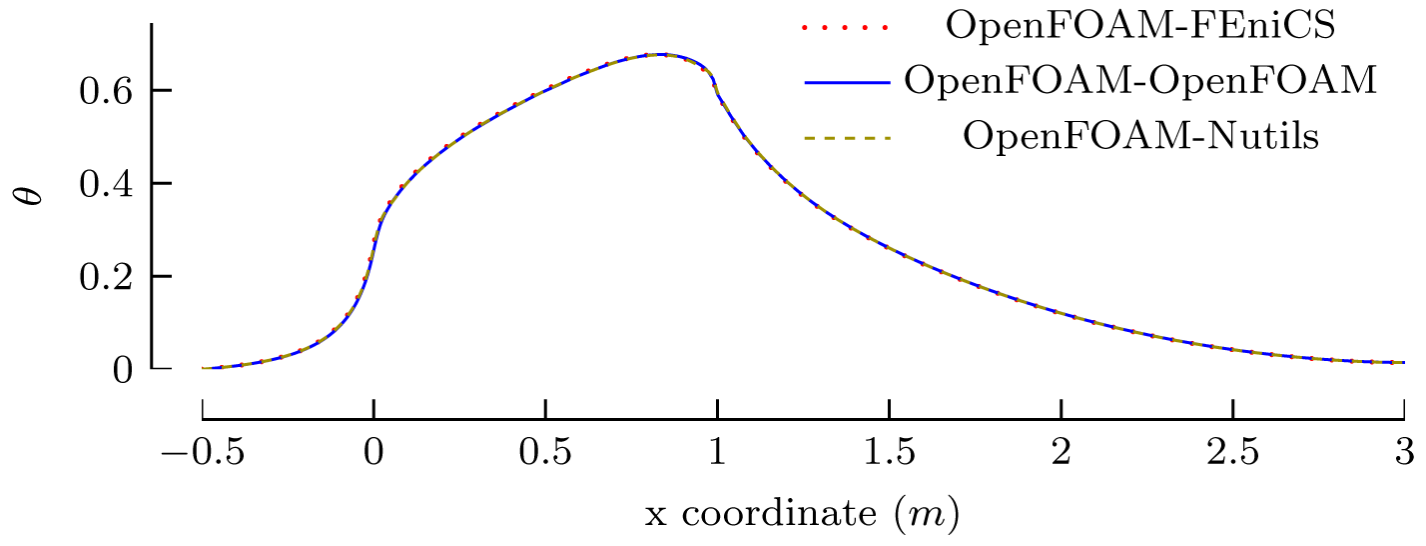
Flow over a heated plate CHT tutorial: Comparison of non-dimensional temperature values *θ* at time *t* = 10 *s* along a line 0.01 *m* above the bottom of the channel for different combinations of solvers. *x* ∈ [−0.5, 0] describes the region of the channel upstream of the plate, *x* ∈ [0, 1] the region where the channel and the plate are coupled and *x* ∈ [1, 3] the downstream region.

A quantity that is commonly monitored in this scenario is the non-dimensional temperature *θ* = ( *T* − *T*_∞_)/( *T _h_* − *T _∞_*) along the interface. Figure 19 depicts identical *θ* profiles for all solver combinations. Furthermore, the isothermal contour plot of the unified fluid-solid domain (cf. Figure 18) is continuous and smooth across the coupling interface. Note that a quantitative comparison to the original work ^98^ is not possible, as our cases describe flow inside a channel and not an open flow.

### 5.2 Flow in a channel with an elastic perpendicular flap FSI tutorial

**Figure 20.**
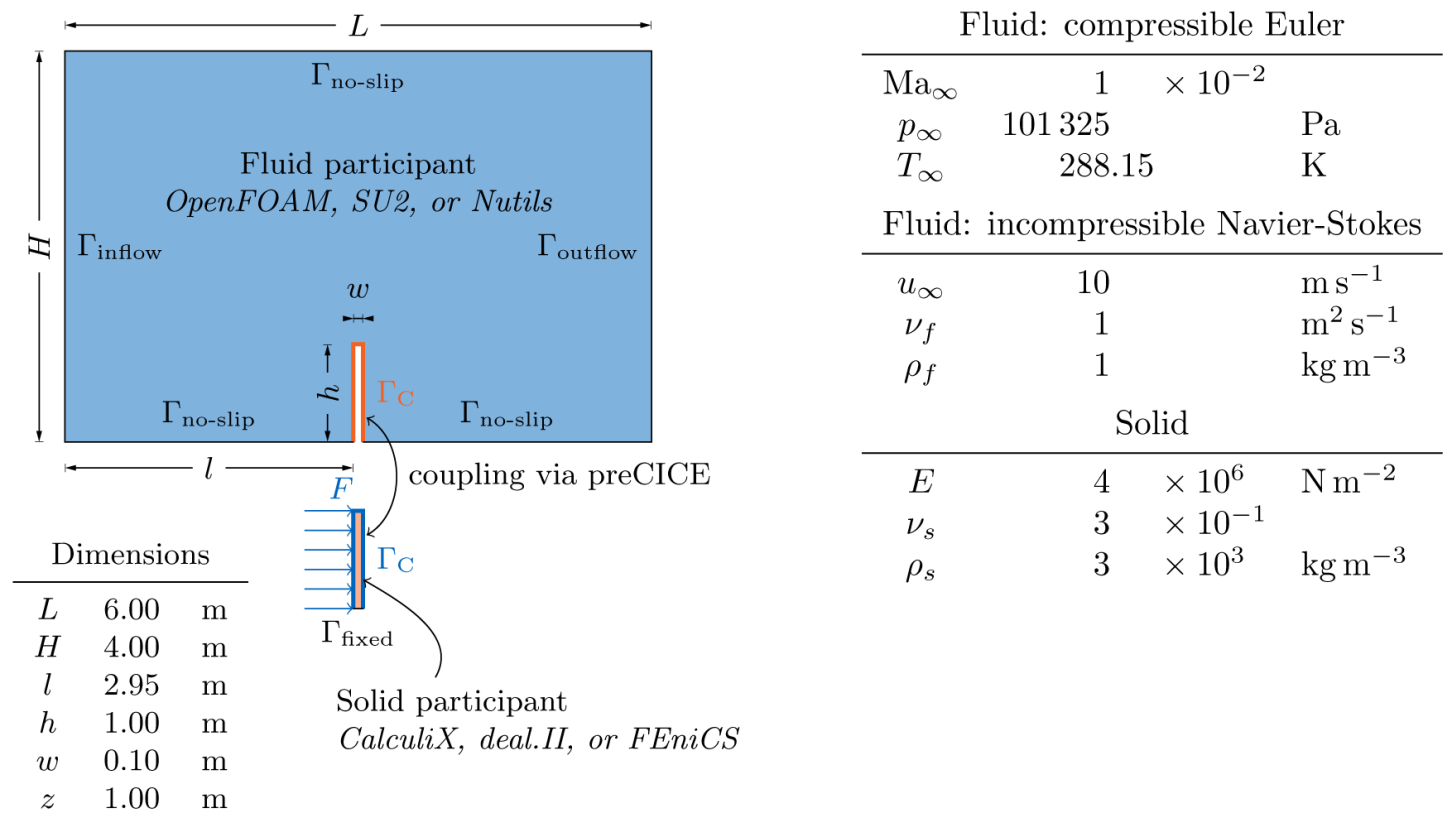
Flow in a channel with an elastic perpendicular flap FSI tutorial: The setup is depicted on the left and physical parameters are listed in the table on the right. The bottom of the flap is clamped, the solid participant reads forces at the interface, while the fluid participant reads displacement values. The inflow velocity at the channel inlet is 10 m/s and the outflow sets a zero velocity gradient. Ma _∞_, *p*_∞_, *T*_∞_, *u*_∞_: Mach number, pressure, temperature, and velocity at the inflow. *ν _f_*, *ρ _f_*: kinematic viscosity and density of the fluid. *E*: Young’s modulus. *ν _s_*: Poisson’s ratio of the solid. *ρ _s_*: density of the solid. *z* is the out-of-plane thickness: even if the coupled case is described as 2D, OpenFOAM and CalculiX are still 3D solvers.

The most common use case of preCICE is FSI. Often, one of the first case users aim to run is the Turek-Hron FSI3 benchmark ^101^. However, this case needs significant computational resources and specifications that are not trivial to achieve with every solver out-of-the-box (e.g., parabolic inlet velocity profile in OpenFOAM). A very common alternative is that of an elastic flap anchored at the bottom of a 2D channel flow as depicted in Figure 20 and further described in the preCICE documentation ^45^.

For the fluid participant, the user can choose between:

1. the incompressible OpenFOAM solver pimpleFoam,
2. an incompressible CFD solver written in Nutils, or
3. the compressible CFD solver of SU2.

For the solid participant, the user can choose among:

1. the linear structure solver of CalculiX with linear, rectangular finite elements,
2. a linear structure solver provided with the deal.II adapter, using fourth order, rectangular finite elements, or
3. a linear structure solver example in FEniCS, using quadratic, triangular finite elements.

All possible combinations ({OpenFOAM, Nutils, SU2} × {CalculiX, deal.II, FEniCS}) use the same preCICE configuration file and the user can select any combination. The fluid solvers read absolute displacement values at the interface (Dirichlet boundary condition) and write forces, while all solid solvers read forces (Neumann boundary condition) and write absolute displacements. By default, the tutorial is configured with RBF data mappings and a parallel-implicit coupling scheme using IQN-ILS acceleration.

**Figure 21.**
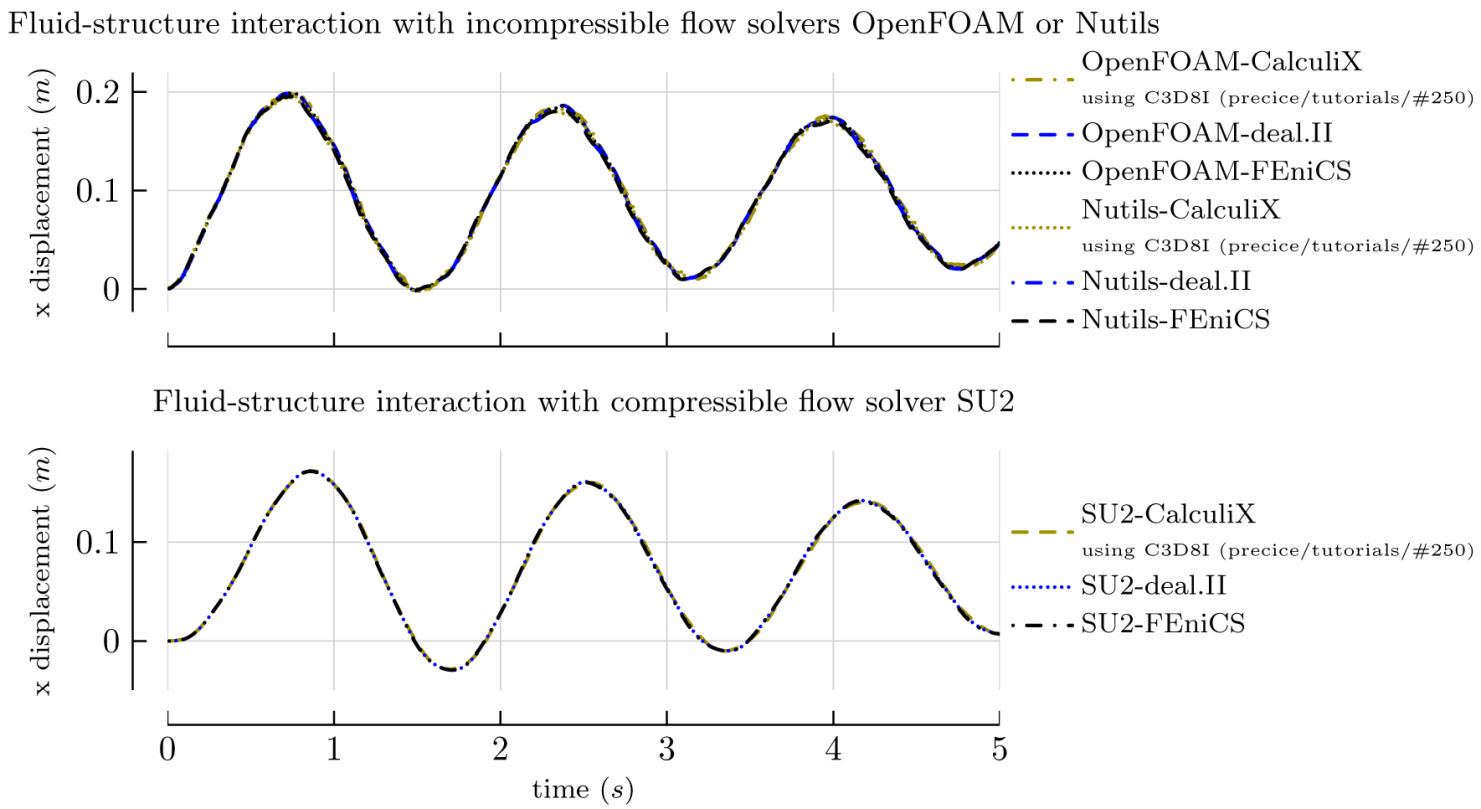
Flow in a channel with an elastic perpendicular flap FSI tutorial: Comparison of the flap tip displacement for different combinations of solvers. The upper plot shows the results for an incompressible flow computed with Nutils or OpenFOAM. The lower plot shows the results for compressible flow computed with SU2. Incompressible and compressible flow give qualitatively different results, as expected. Good agreement within each class of flow simulation is achieved for all combinations of solid and fluid solvers. When using CalculiX as solid solver, the distribution v2104.0 ^19^ specified C3D8 elements, which led to insufficient agreement with the results of the rest of the solvers. Using C3D8I elements produces results which are in better agreement, a suggestion contributed after the release of v2104.0 by Andrés Pedemonte Fehrmann ( https://github.com/precice/tutorials/pull/250) and used for the results shown here.

**Figure 22.**
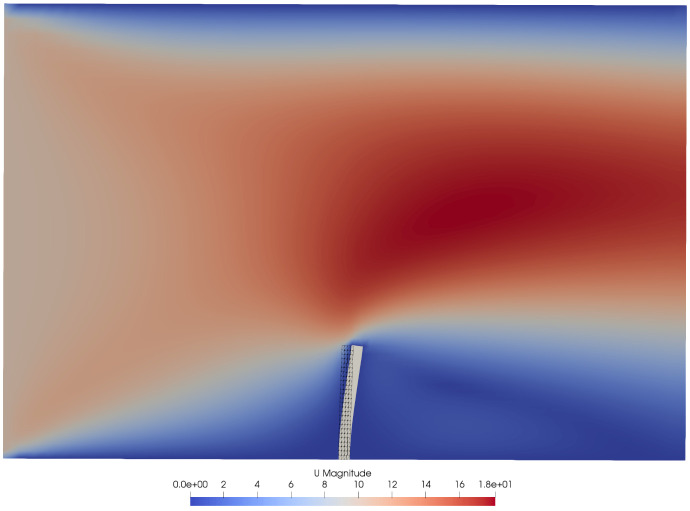
Flow in a channel with an elastic perpendicular flap FSI tutorial: Visualization of flow field and deformed flap at time *t* = 2 *s* using OpenFOAM as fluid and FEniCS as solid solver. For comparison, the deformed solid mesh of an SU2-FEniCS simulation is shown in black to make the difference between FSI with a compressible and an incompressible fluid simulation visible.

A quantity that is commonly monitored in this scenario is the displacement of the tip of the flap. We track this quantity using a preCICE watchpoint and compare the results across different solver combinations in Figure 21. Figure 22 shows a direct comparison snapshot of an FSI simulation with an incompressible and a compressible fluid solver.

## 6 Testing and continuous integration

The core preCICE library and the non-core components are developed in separate repositories, each with a number of continuous integration (CI) workflows. These workflows are tests, quality assurance checks, or operations to prepare and validate packages. We find such workflows to be indispensable for multi-component, multi-developers projects, as they answer questions such as ’will the code still compile and behave in the same way if we integrate these changes?’, ’will a simulation still give the same results?’, and ’will these changes have side-effects in other (potentially not regularly updated) components?’. In other words, these workflows facilitate further development by ensuring that everything still works.

**Figure 23.**
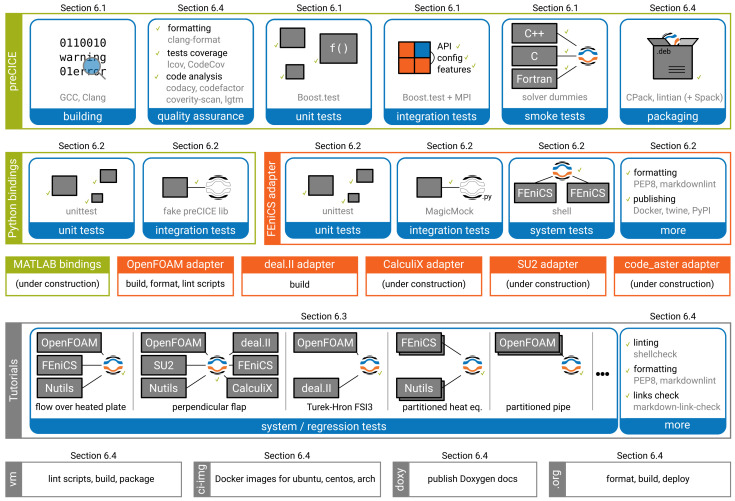
Overview of the testing and continuous integration workflows for different preCICE components. Each box represents a separate project repository. The non-native language bindings depend on preCICE. The adapters depend on language bindings or directly on preCICE. The tutorials and system tests depend on all the adapters. The components at the bottom-most row (virtual machine image, CI images, Doxygen documentation, website) depend on one or more other components. Read more details about each workflow in the section number listed above it.

Figure 23 depicts the currently deployed workflows. As one may observe, the granularity of testing and CI correlates to the number of users and developers involved in each subproject. In some cases, it may also be enabled or hindered by the respective programming language environment. As the most important and actively developed component, the core library is rigorously tested in a wide range of levels. The rich tooling collection of Python enables the continuous integration (CI) of the Python bindings and the FEniCS adapter, while we are gradually adding similar workflows to the rest of the adapters. The tutorials provide a platform to test every component in complete simulations (system tests with results regression checks). Finally, a few additional workflows keep non-critical systems up-to-date.

The number and diversity of components required to construct a complete coupled simulation (at least two participants + multiple components per participant), as well as the challenges in testing each component in isolation, makes testing a coupling library significantly more complex than testing a linear algebra solver library, for example. Complex testing approaches of significant novelty are required. We structure the rest of the section following the different complexity levels. In Section 6.1, we present the CI of the core library, for which no interaction with other components is required. In Section 6.2, we continue with the CI of non-native language bindings and adapters. This layer depends on the core library, as well as on external components (the solvers), leading to a need for testing in isolation. In Section 6.3, we construct system tests for the complete software stack. Finally, in Section 6.4, we give an overview of additional checks and workflows which we use across the whole project.

### 6.1 Tests for the preCICE core library

To test the complete functionality of the preCICE core library, heterogeneous test setups are needed. Individual tests may require one or more logical participants running on one or more message passing interface (MPI) ranks. To solve this intrinsic problem of testing a communication and orchestration library in a parallel environment, the core library tests are run on four MPI ranks. Partitioning these four ranks allows to cover various scenarios, from testing math functions on a single rank, over testing parallel mappings on three ranks, up to testing scenarios with a serial participant on a single rank coupled to a parallel participant on three ranks. As mature MPI-aware testing frameworks are not available, we developed our own testing framework extending Boost.test to support the aforementioned criteria.

**Figure 24.**
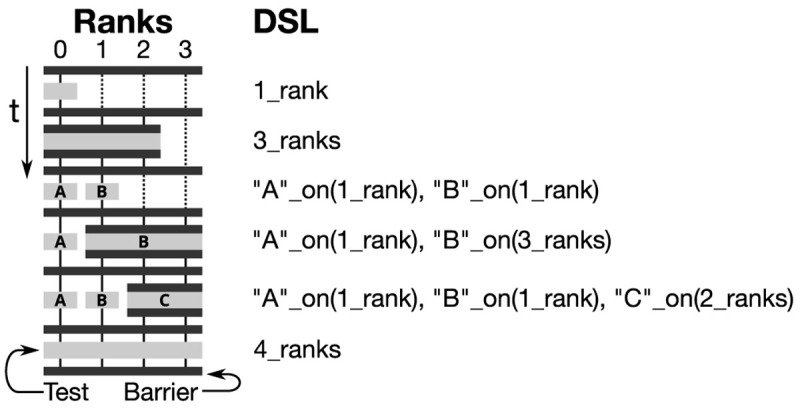
This figure depicts the MPI communicator setup on the left corresponding to a sequence of test setups on the right. The testing framework uses four MPI ranks, depicted by the vertical lines. Horizontal black bars are barriers and dotted lines are ranks which are unused during a test setup and hence idle. Each test starts with PRECICE_TEST(...), containing an expression based on the DSL, which results in a complete test setup. The test DSL specifies the name of local participants followed by the amount of ranks. The name is optional for tests containing only a single participant.

The extension of Boost.Test provides a custom domain-specific language (DSL), which is used to set up a PRECICE_TEST(). The DSL specifies the name of local participants used in the test, followed by the amount of ranks and optional requirements. If a test contains only a single participant, then its name can be omitted. The DSL is human-readable, examples are "A"_on(2_ranks), "B"_on(1_rank) or simply 1_rank. The implementation of the DSL firstly restricts the MPI communicator size to the required amount of total ranks, followed by grouping ranks by name, forming communicators for the logical participants. Further specified requirements on logical participants, such as initialization of sub-components, are then handled inside the isolated state of each participant. The result of each PRECICE_TEST() is an immutable object which, for each test rank, provides access to the context including name and communicator information of the local participant. The context holds further information about the setup, information which allows to sanitize user input provided to utility functions. At the end of each test, the context object firstly reverts all changes made by setup requirements, secondly ungroups the communicators, and finally synchronizes all ranks, including ranks not needed by the test. See Figure 24 for example configurations of this framework.

The core library tests can be categorized into unit tests, integration tests, and code-example tests. Currently, preCICE is tested with a total of 438 unit and integration tests.

**Unit tests** This type tests a component in isolation, using its public interface. The test functions manually set up the majority of required components and partition the available ranks according to the needs of the test. The needs for partitioning vary: First, many unit tests handling geometric functions, VTK exports, and mesh internals require only a single logical participant and run on a single rank. Furthermore, components such as VTK exports and radial-basis-function mappings have additional functionality when running in parallel, hence require multiple ranks on a single logical unit. Finally, inter-code communication, the coupling schemes, and the mesh partitioning require multiple logical participants. Each of these logical participants may run on one or multiple ranks. See Listing 5 for an example of a unit test.



 1 BOOST_AUTO_TEST_CASE(ParallelMappingTest)
 2 {
 3  PRECICE_TEST(""_on(4_ranks).setupMasterSlaves(), Require::PETSc);
 4  constexpr int dims = 2;
 5  // Setup InMesh
 6  mesh::PtrMesh inMesh(new mesh::Mesh("InMesh", dims));
 7  mesh::PtrData inData = inMesh->createData("InData", dims);
 8  getDistributedInMesh(context, inMesh, inData);
 9  // Setup OutMesh ...
10  // Setup Mapping
11  PetRadialBasisFctMapping<Gaussian> mapping{
12    Mapping::CONSISTENT, dims, Gaussian{5.0}};
13  mapping.setMeshes(inMesh, outMesh);
14  // Test the Mapping preparation
15  BOOST_TEST(not mapping.hasComputedMapping());
16  mapping.computeMapping();
17  BOOST_TEST(mapping.hasComputedMapping());
18  // Test the Data Mapping
19  BOOST_TEST(not mapping.hasComputedMapping());
20  mapping.map(inData->getID(), outData->getID());
21  BOOST_TEST(outData->values() == expectedData);
22 }


**Listing 5.** Example unit test of a parallel RBF mapping running on four ranks. Further requirements on the test are the setup of the master-slave communication and the initialization of PETSc. First the test defines meshes and associated data followed by setting up and executing the mapping. The result is then checked against the expected outcome. The example showcases the preparation involved in testing individual components of preCICE in a parallel context.

**Integration tests** This type uses the application programming interface (API) of preCICE itself to test specific scenarios, hence the test setup is handled using a preCICE configuration file. Individual logical participants may run on a single rank each, to test coupling of serial solvers with various setups. Another very common setup consists of two logical participants running on two ranks each. This allows to thoroughly test the partitioning behavior given various mapping schemes. Integration tests are also used to reproduce and fix bugs reported by users. See Listing 6 for an example of an integration test.


 1 BOOST_AUTO_TEST_CASE(ParallelIntegrationTest2x2)
 2 {
 3  PRECICE_TEST("SolverOne"_on(2_ranks), "SolverTwo"_on(2_ranks));
 4  std::string meshName, writeDataName, readDataName;
 5  if (context.isNamed("SolverOne")) {
 6   meshName    = "MeshOne";
 7   writeDataName = "Data1";
 8   readDataName = "Data2";
 9  } else {
10   // ...
11  }
12  SolverInterface interface(context.name, _pathToTests + "test-config.xml",
13    context.rank, context.size);
14  // set mesh vertices and initialize
15  std::array<double, 4> inValues, outValues;
16  while (interface.isCouplingOngoing()) {
17   interface.readBlockScalarData(readDataID, 4, vertexIDs, inValues);
18   if (context.isNamed("SolverOne")) {
19    outValues = inValues;
20   } else {
21    outValues = solveSystem(inValues);
22   }
23   interface.writeBlockScalarData(writeDataID, 4, vertexIDs, outValues);
24   interface.advance(1.0);
25 }
26 BOOST_TEST(outValues == expectedValues);
27 }



**Listing 6.** Example integration test involving two parallel participants SolverOne and SolverTwo running on two ranks each. The context object provides information about the identity of the current rank. This information is used to setup further local information such as mesh and data names. Integration tests then directly construct a SolverInterface and use preCICE API calls to run the test.

**Code-example tests** This type smoke-tests native bindings using the provided examples. Native-bindings are C and Fortran bindings, which are implemented using the C++ API of preCICE and linked directly into the library. Non-native bindings such as Python are covered in Section 6.2. Each language binding comes with an example program called a *solverdummy*. All solverdummies implement the same functionality and provide a template for using the preCICE API in the respective programming language. The code-example tests themselves consist of three steps: First, the tests build each solverdummy and link it to the preCICE library. This tests a common subset of the interface of the bindings for completeness and assures that the build system is functional. Second, they run a small coupled simulation coupling each solverdummy to itself. This ensures that the used language binding is working correctly. Finally, they run a small coupled simulation coupling different solverdummies to each other. This ensures that the bindings (of different languages) are compatible.

### 6.2 Tests for adapters and bindings

As explained in Section 3.4 and Section 4, language bindings and adapters are organized in independent repositories. The requirements for tests of such non-core components are identical to the ones for the core library: the non-core components must also comprise of valid code, their individual units should behave in the correct way and work together, while continuous integration tests should be performed on every commit to each component repository. We again distinguish unit tests and integration tests.

**Unit tests** We do not need a special treatment for unit tests of non-core components. Note that non-core components are written in various languages. This requires the use of suitable testing frameworks for each language, such as the Python module unittest for the Python bindings and the FEniCS adapter.

**Integration tests** Non-core components use preCICE through its API, treating it as a regular, black-box dependency. This safeguards low software coupling, but also leads to a technical complication as already mentioned above: Due to the very nature of coupled simulations, preCICE requires at least two participants for executing most steps of a simulation. This cannot be avoided easily (since the components are not able to modify preCICE itself) and, therefore, it is not trivial to test each component independently.

To solve this problem we use a strategy commonly known as *mocking*. This is a well-known and widely established software engineering practice ^102^, but not as widespread in the scientific software community. Mocking is useful, if the system under test (non-core component) has another component (preCICE) as a dependency and interacts with this component through its API. Since we want to avoid starting a second participant in our integration tests, we use a mocked version of preCICE instead of the original one. This mocked preCICE returns fake output for testing and does not rely on any other components. In the following, we give two examples for our implementation of this testing pattern: first, integration tests in the FEniCS adapter and, second, integration tests in the Python language bindings of preCICE. In both cases, API calls to the fake version of preCICE do not require any initialization of a second participant and hard-coded fake values are returned. This allows to write short and simple tests.

Mock testing for the FEniCS adapter heavily relies on the Python module unittest.mock, which allows creating a MagicMock object that is used to provide a fake implementation of functions or objects. Additionally, the module provides a patch function that allows to replace a module that a test imports with a fake version of the same module, at runtime. These two pieces allow us to replace the Python bindings of preCICE with a fake version. For a detailed example, please refer to the adapter reference paper ^86^.

Mock testing for the Python bindings is more involved, since the bindings rely on two different languages, C++ and Python, and (to the authors’ knowledge) no mocking framework exists for this purpose. We, thus, test the Python bindings by building a specific executable, where we link against the mocked version of preCICE: a single SolverInterface.cpp with a fake implementation. We do include the original interface of preCICE ( SolverInterface.hpp) to make sure that the API is consistent. This allows us to keep the application code of the Python bindings clean and to decide whether we want to use the real or fake implementation of the preCICE library at compile time. The integration tests of the Python bindings then allow us to check for the correctness of type conversions done by the language bindings, such as converting a C++ double* array to a numpy array ^103^ using Cython ^104^. An example is given in Listing 7. Our mocking approach leads to a non-standard setup.py build script, which allows us to choose whether we want to build the real executable or the one intended for testing through the standard interface of setuptools ^46^. A nice side effect of this testing pattern is that preCICE itself is not even needed and does not have to be installed on the system running the tests.


void setMeshVertices(int meshID, int size, const double *positions, int * ids){
 std::vector<int> fake_ids = get_hardcoded_ids(size);
 std::copy(fake_ids.begin(), fake_ids.end(), ids);
}


**Listing 7.** Fake implementation of setMeshVertices used for integration tests of Python bindings. The fake version of setMeshVertices just returns fake vertex IDs. If an integration test of the Python bindings is calling this function, one can easily check whether the obtained vertex IDs are the expected ones using Python’s unit testing framework.

**Outlook** Testing of other language bindings and adapters is under current development: Our prototype for integration tests for the OpenFOAM adapter uses the mock testing pattern and the C++ mocking framework FakeIt ^47^. For testing the MATLAB bindings, the existing Python bindings testing approach may be used.

### 6.3 System and regression tests

Fine-grained unit and integration tests can give us detailed insight into each component, but these tests only study each component or group of components in isolation. System tests give us the user perspective of all components working together: ‘does the coupled simulation black-box still behave in the same way?’ and ‘if not, which change in which component introduced the regression?’. While system tests can be quite straight-forward in their implementation, developing effective system tests for multi-component, multi-participant simulations becomes a complex task, especially when considering different stakeholder perspectives.

Let us look at a few examples of such stakeholder perspectives. As a release *manager*, ***Ma**ria* wants to know that the latest state of all development branches to be released works flawlessly together on the release day, so that she can release a new version. As a developer of the core *library*, ***Li**sa* wants to know that her proposed changes do not cause any unintended regression in results or behavior in the context of a complete simulation. As a developer of an *adapter*, ***Ad**am* has even more questions. First, similarly to Lisa, he wants to know that his proposed changes do not cause any regressions downstream. Additionally, Adam wants to know if he needs to update his adapter to support breaking changes (of installation/configuration) in the development branches of upstream components or new solver and dependency versions. As a developer of *tutorial* cases, ***Tu**dor* wants to know that configuration updates do not cause regressions and that the tutorials still work as expected with newer solver and dependency versions. Finally, as a maintainer of the *system tests*, ****Sy**** wants to know that their proposed changes do not cause any downtime to the operations.

**Figure 25.**
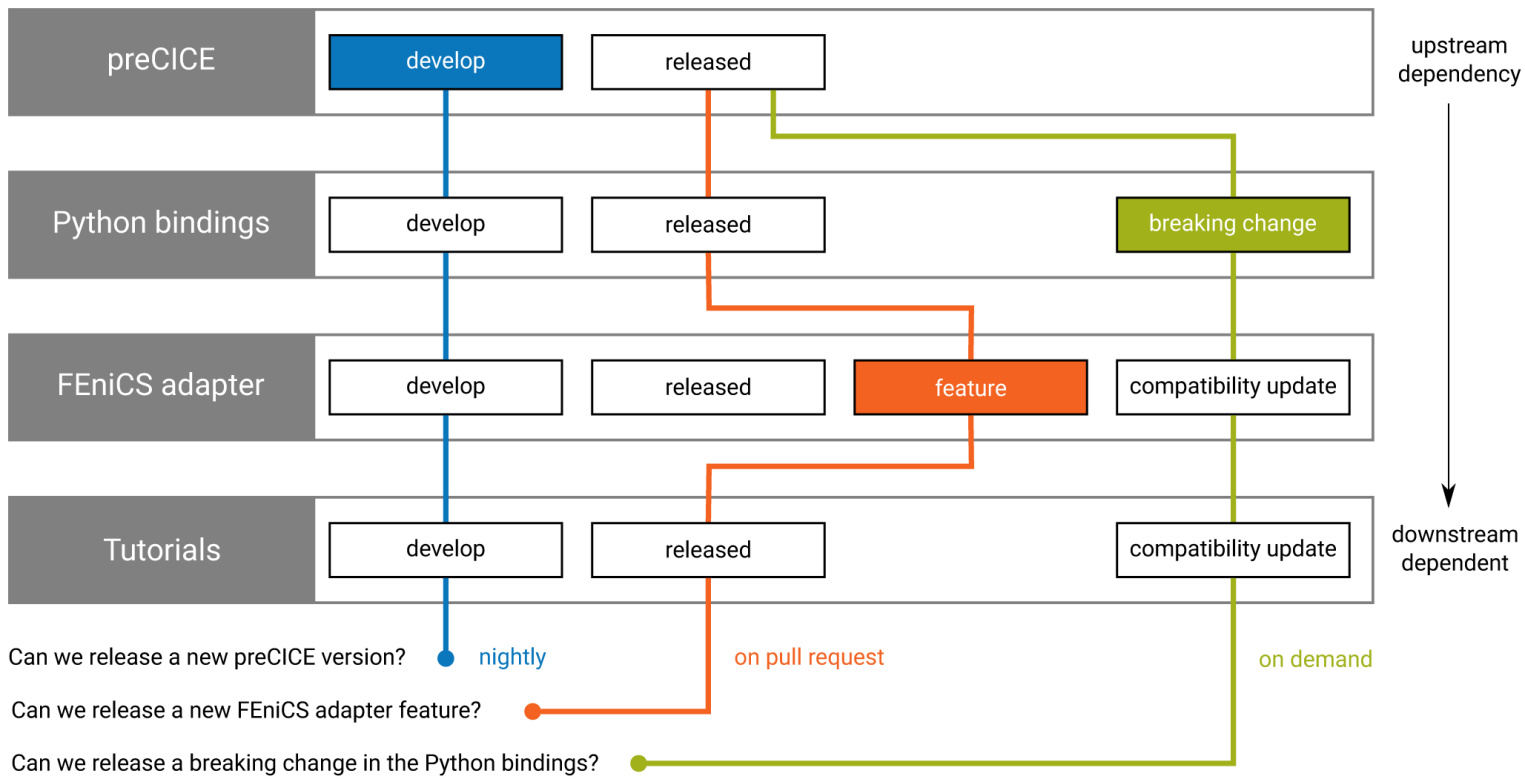
Example questions that the system (regression) tests of preCICE help us answer (planned workflow). The system tests are always executed at the bottom-most layer (tutorials repository), using Docker images prepared in the layers above. Every night, all develop branches are tested together, to ensure that a new version of preCICE can be released at any time (blue). Pull requests that introduce non-breaking changes are automatically tested against the latest released versions of the other repositories (orange). Pull requests that introduce breaking changes need additional coordination with downstream projects and the tests need to compose compatible branches (green). The Python bindings and the FEniCS adapter only serve here as examples; in practice, more repositories are involved.

The situation we just described becomes apparent looking at Figure 25. The test matrix evolves into a cross product of: 


{platform}×{preCICEbranches}×{bindingsb.}×{adapterb.}×{tutorialb.}×{systemtestsb.}

As these tests take a long time to prepare and execute and as they are particularly challenging to log in a structured and effective way, executing the complete test matrix is not realistic and we need to select representative configurations.

We restrict the test matrix to a set of strategically important combinations. In terms of platforms, we execute most tests on the platform most common among users, currently the latest Ubuntu LTS version 20.04 ^48^. We also execute selected tests on the oldest supported platform (previous Ubuntu LTS) and on the latest state of the continuously-updated Arch Linux ^49^. In terms of branches, we test each proposed branch with the rest of the components in their latest released state: this helps Lisa, Adam, and Tudor develop their projects independently, without worrying about untested new features. In case a breaking change is introduced in one component, then this needs to be tested in combination with corresponding compatibility updates in the downstream components. We also test the development branches of all components together in nightly builds, so that Maria and every developer can confidently release new versions of each component.

Maintaining the system tests and reference data up-to-date requires significant effort and any failing tests need to be addressed quickly, so that they remain useful and trusted. We observed that deep understanding and documentation of known issues that trigger test failures is crucial for the infrastructure to facilitate instead of hinder the development. Similarly, easily accessible logging of different levels is very important to verify that all relevant tests have succeeded and to precisely identify any faults. Finally, even though the operations need to be automatic enough to get green lights at the right places, developers do want to be able to form a clear mental model of the system behind the automation in order to trust the system and try to debug it, if needed.

Since preCICE v1 and till v2.1, we maintained the system tests on a dedicated repository ^50^ using Travis CI. This repository contained scripts to run tests, scripts to prepare the test cases, as well as reference data for each test case. Because of policy changes in Travis CI and increasing flexibility offered by newer alternatives, we phased-out this implementation and we have been migrating to a different system. We describe here the outdated architecture of the tests used for preCICE v2.1 and discuss issues and potential solutions.

With every new commit pushed to an adapter repository, the Travis CI instance of the adapter instructed the Travis CI instance of the systemtests repository to run any tests (tutorials) it knew to involve this adapter. Travis CI then built Docker images of preCICE adapters and pushed these to Docker Hub so that they could be reused. It then started one Docker container per simulation participant, as well as one Docker container serving the tutorial configuration. We used Docker Compose to build connections between the containers and we set a commonly accessible directory to exchange necessary connection tokens for the inter-code communication, as described in Section 3.3.

At the end of each test case, a script compared the results. We originally compared every available results file excluding lines unique to every run. To account for sporadic rounding errors, we filtered arithmetic data and applied a numerical comparison. As this approach was very tedious to maintain for every new solver, we switched to comparing only the exported VTK files of the preCICE interface meshes. With a common file format at hand, the comparison scripts became much easier to execute and maintain. We also found this simplification to be enough for identifying regressions in the coupling, which is our main interest. After Travis CI compared the results, it archived key log files to a dedicated repository precice_st_output. This was a far-from-ideal logging solution, leading to cumbersome workflows to discover more details about the executed tests and potential failures.

We are currently redesigning our system tests. Key decisions so far have been to separate the machinery from the reference data, hosting the (reduced) reference data together with the configurations that produce them (tutorials), so that they can be updated at the same time. More recent tools, such as GitHub Actions ^51^ and GitLab CI ^52^, offer multi-project pipelines and more possibilities for storing artifacts and archiving logs. With such additional options to avoid complex workarounds and with our experience from the aforementioned approaches, a redesigning was deemed reasonable and is expected to fruit in the near future. Until then, we rely on regular manual runs with every release.

### 6.4 Additional checks

To maintain the quality and consistency of the codebase, the preCICE CI runs additional checks on the latest state of every pull request. The CI uses a fixed version of clang-format ^53^ to check all C++ and C files, as well as a custom formatter based on the Python lxml ^54^ package to check all XML configuration files for correct formatting. As most CI environments provide multiple CPUs, the system uses GNU parallel ^105^ to leverage the available compute resources.

When building and testing on Ubuntu, the CI additionally generates the Debian packages using CPack ^55^ and test them using the Debian package checker Lintian ^56^. Furthermore, the tests generate code testing coverage information using the --coverage option of the GNU C++ compiler. The resulting coverage information is then gathered by LCOV ^57^ and uploaded to the Codecov ^58^ service, which integrates the coverage report into the GitHub user interface. This informs the reviewer about the coverage of the code change, as well as the resulting coverage change of the whole project.

Moreover, the external code quality services lgtm ^59^, CodeFactor ^60^, and Codacy ^61^ are integrated into the core library’s GitHub project and automatically run to perform code analyses using webhooks. A scheduled job runs the proprietary static code analysis tool Coverity Scan ^62^ on the codebase once per week and reports the results to the developer mailing list. These tools are useful to find less obvious issues such as code complexity, code duplication, misspellings as well as technical issues such as unreachable code, code paths leading to using uninitialized variables, incorrect exception handling, and more.

We use publicly available GitHub Actions from the marketplace ^63^ to apply such checks on less critical components: we validate shell scripts using shellcheck ^64^, we validate and format Python scripts using autopep8 ^65^, we validate the syntax of our documentation files with markdownlint ^66^, and we check for broken hyperlinks using markdown- link-check ^67^. Finally, we publish Python packages using twine ^68^.

Code reviews provide an additional safety check, which can prevent issues that are otherwise difficult to check automatically. Pull request templates provide checklists for authors and reviewers. Most non-trivial code contributions to preCICE since 2018 are reviewed by at least one further core developer. The master branches are protected from pushing and from merging without reviews.

Apart from tests and quality checks, a few more operations contribute to maintaining the resources available to the user up-to-date. A GitHub Actions workflow builds and packages a Vagrant ^69^ box for VirtualBox with the latest Ubuntu LTS and all common components and tutorials pre-installed. A similar workflow prepares and publishes Docker images with all the preCICE dependencies, images which we use for our CI ^70^. The website of preCICE is also automatically generated using GitHub Pages ^71^, integrating content from additional repositories (tutorials and adapters). Finally, a dedicated workflow periodically updates the Doxygen-based C++ source documentation.

## 7 Community

As the purpose of preCICE is to connect different simulation software, preCICE naturally also helps connecting researchers – imagine the fluid mechanics group and the solid mechanics group of a computational mechanics faculty with their individual in-house CFD and FEM codes. In the past five years, a significant community of users has been formed around preCICE, with some of them also contributing back code or tutorials. The *preDOM* project ^72^, funded by the German Research Foundation, played an important role for this development. In fact, most of the improvements described in this paper were part of the project: building and packaging, adapters, tutorials, tests, and continuous integration, but also user documentation and community building.

**Figure 26.**
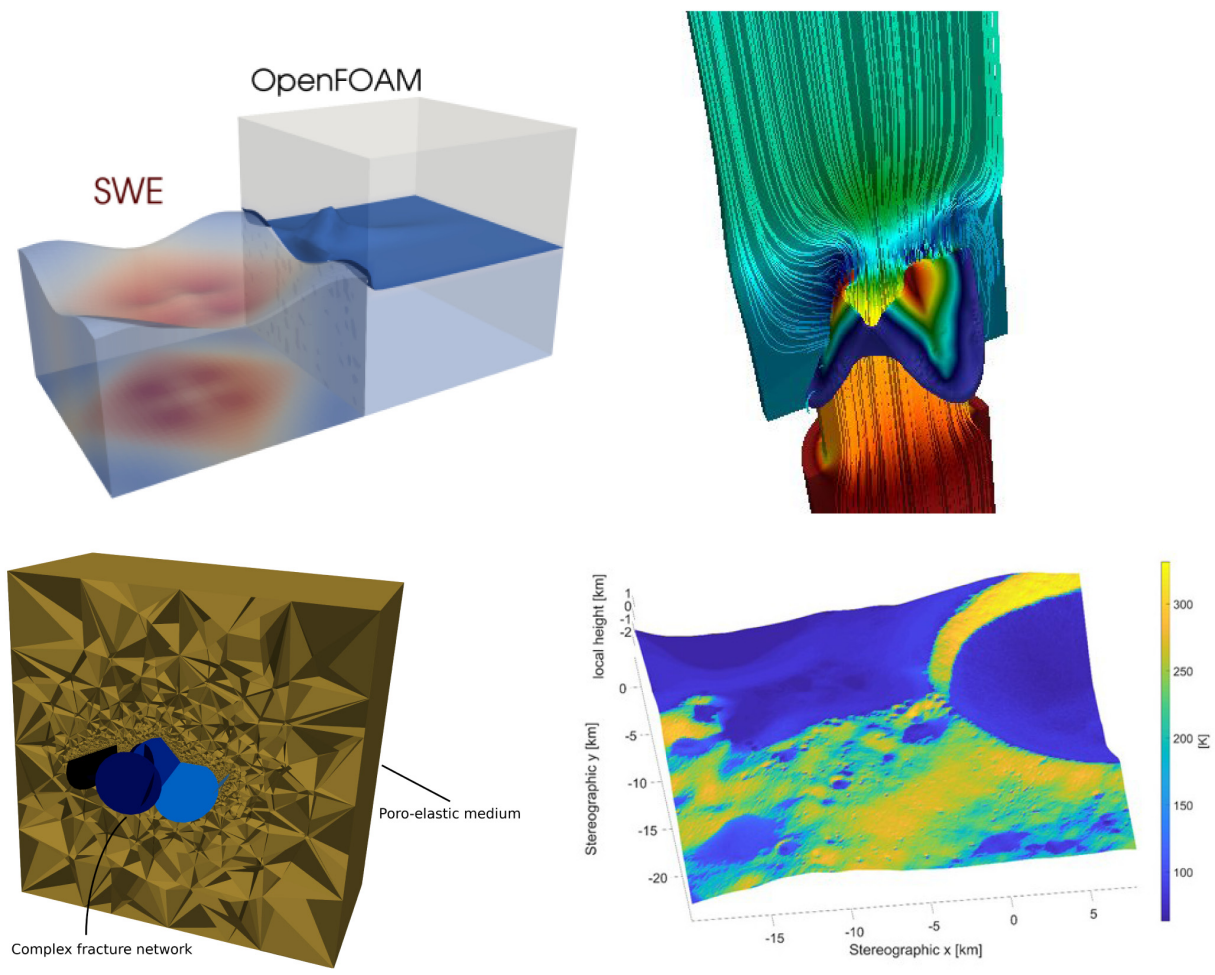
Various simulations from the preCICE community. All pictures taken from the page *Community stories* on precice.org. Top left: a shallow-water equations solver coupled to OpenFOAM ^124^. Top right: an artificial heart valve simulated with OpenFOAM and CalculiX ^115^. Bottom left: A 3D poro-mechanics model coupled to 2D fluid equations, both implemented in FEniCS ^119^. Bottom right: a MATLAB heat equation solver coupled to a GPU ray-tracing software package to simulate heat conduction and radiation on the surface of the moon ^59^.

Today, we know through forum discussions, conferences, workshops, and publications of more than 100 research groups using preCICE. Roughly one half of them are from academia, while the other half comes from non-academic research centers (e.g., the German Max Planck Institute for Plasma Physics, the German Helmholtz-Zentrum Hereon, the Italian Aerospace Research Centre, or A*STAR in Singapore) or industry (e.g., MTU Aero Engines or Bitron). We collect some user stories on our website ^73^ and depict some highlights in Figure 26. Presumably half of the users apply preCICE for fluid-structure interaction or conjugate heat transfer applications. The other half uses preCICE for more uncommon setups, for example, coupling of different fluid models with each other ^106^ or coupling of CFD to particle methods ^11^.

**Applications and Software** A non-exhaustive list of application fields includes mechanical and civil engineering (astronautics ^59^, manufacturing processes ^66, 69^, aerodynamics ^23, 64. 107– 111^, urban wind modeling ^106^, aeroacoustics ^6, 112^, explosions ^113, 114^), marine engineering ^22^, bio engineering (heart valves ^115^, aortic blood flow ^7^, fish locomotion ^116^, muscle-tendon systems ^10^), nuclear fission and fusion reactors ^9, 117, 118^, and geophysics ^8, 12, 119^. Many users do not only use the official adapters (cf. Section 4), but couple further community codes or their in-house codes. A non-exhaustive list of available coupled software (under a commercial or an open-source license) includes CAMRAD II and TAU ^110^, DUST ^23^, DuMuX ^8, 120^, DUNE ^121^, Rhoxyz ^22^, Ateles ^122^, XDEM ^11^, and FLEXI ^123^.

**Figure 27.**
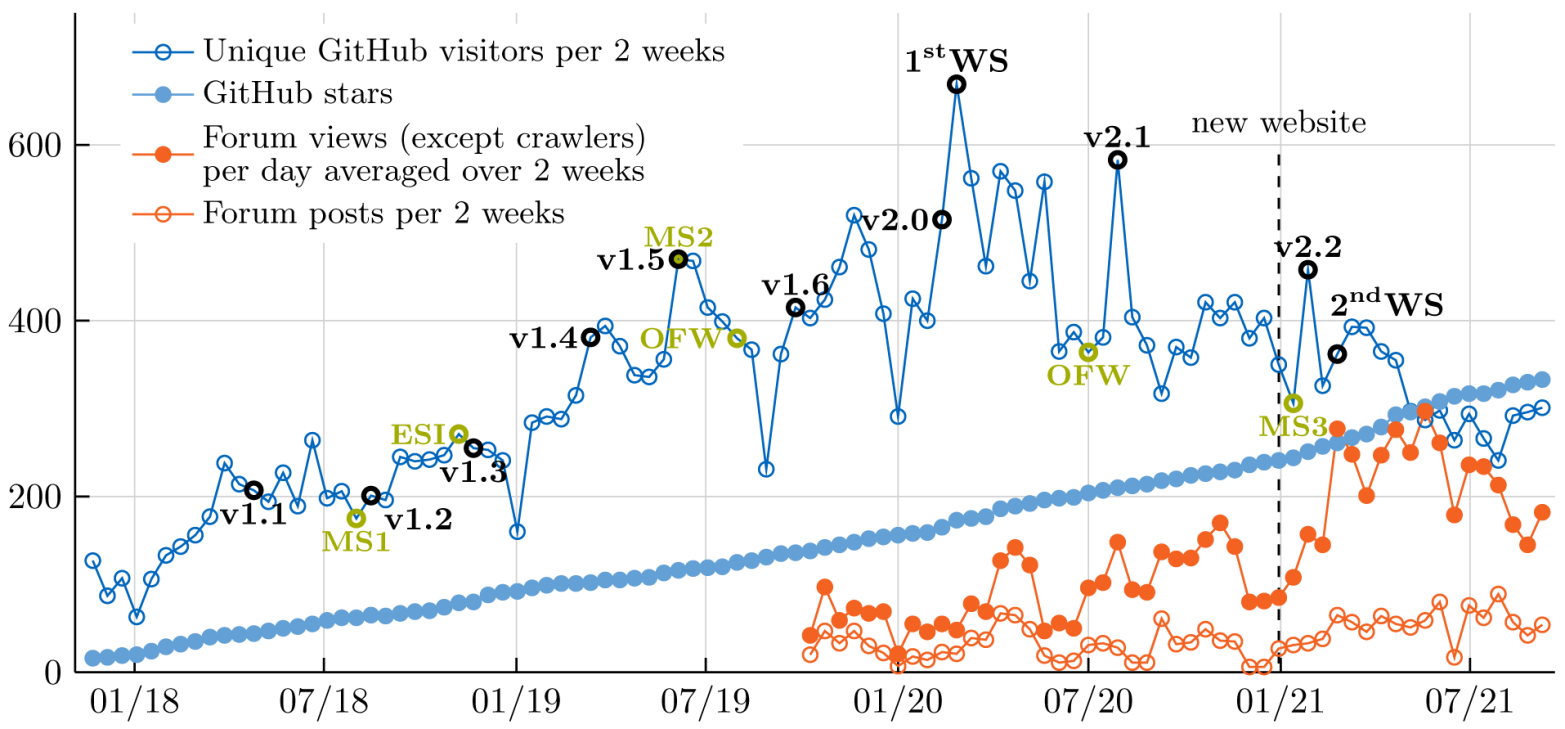
Various traffic data showing community growth over time connected to key events. Whereas new releases have a clear impact on GitHub traffic, conferences, such as the ECCOMAS mini-symposia (MS1, MS2, MS3), different OpenFOAM workshops (ESI, OFW), and the preCICE workshops (WS) have not always a clear direct impact. With the start of the preCICE forum (fall 2019) and, in particular, with moving the user documentation to the new website at the end of 2020, traffic is shifted away from GitHub. At the online preCICE Workshop 2021, we used the forum to let attendees introduce themselves. This led to sustainable increase of forum traffic.

**Community Building** preCICE users can interact with developers and with each other through various channels. We provide and moderate a Discourse forum ^74^ and a Gitter chat room ^75^. The forum replaced a previously used mailing list as discussions in the forum can be much better structured through categories, labels, and *solution* posts. Moreover, Discourse can be customized to great extent, which allows us to hand over moderation responsibilities to the community at a suitable pace. For feature requests and bug reports, we use the issue trackers of the different repositories on GitHub ^76^. Moreover, we organize yearly mini-symposia at ECCOMAS conferences (ECCM-ECFD 2018, COUPLED 2019, WCCM 2020, COUPLED 2021) and our own preCICE Workshops (preCICE Workshop 2020 in Munich ^77^, preCICE Workshop 2021 and 2022 online). The workshops include an introduction course, which we plan to further extend in the next years. Figure 27 shows a static growth of the preCICE community over the previous three to four years.

**Contributions** To strengthen the sustainability of preCICE, we encourage users to also contribute back. Example contributions encompass code, tutorials, bug reports, or documentation. On our website, we provide detailed contributing guidelines ^78^ while every external contribution is reviewed for functionality, coding practices, consistency, and usability. Our long-term goal is to hand over development of the official adapters (cf. Section 4) to the community. In recent years, the OpenFOAM adapter has, in particular, seen various external contributions ^125^ and serves as an example of how the community may successfully contribute to isolated, smaller *compartments* of a software project, as they can be easier to understand and contribute to. As of July 2021, 20% (18 out of 89) pull requests and 26% (26 out of 100) issues in the OpenFOAM adapter repository have been contributed by external contributors (not from the academic groups of the core team). While half of the external pull requests were ultimately not merged, they still serve as proof of concept for features that were at the time not aligned with the direction of the project. We have observed that several non-merged contributions were still useful for the community and we expect that tooling, automation, and clear guidelines will increase the ratio of successful external contributions in the long run.

## 8 Conclusions

We have shown on the basis of various aspects that there is a tremendous gap between a working prototype software – a software with state-of-the-art numerical and HPC methods (preCICE in 2016) and a sustainable and user-friendly software (preCICE in 2021). While the first one allows for scientific discoveries in scientific computing, only the latter allows for scientific discoveries in application areas as well. This can also be observed in the user numbers of preCICE. While the software today has a large and vivid community of users in a wide variety of application areas, it hardly had any users in 2016. To bridge this gap, we presented necessary efforts in documentation, building, packaging, integration with external software, tutorials, tests, continuous integration, and community building. Nearly all of these aspects are more complicated for a multi-component coupling software such as preCICE than for most other scientific computing software. This is not only due to the fact that preCICE is a library and, thus, needs another program that calls preCICE, but also that a coupled simulation needs by definition at least two different programs to be coupled. Therefore, often novel solutions are necessary for usually standard problems, such as the variety of testing concepts introduced in Section 6.

In forthcoming years, preCICE will undergo various extensions to make the software applicable beyond low-order, mesh-based, surface-coupled problems, such as fluid-structure interaction. Current work focuses on geometric multi-scale coupling (especially 1D-3D and 2D-3D mapping), dynamic coupling meshes, waveform iteration ^40^, mesh-particle coupling, macro-micro coupling, and coupling to data-based approaches. An important topic will also be the efficient support of volume-coupled problems, which requires novel ideas in all main ingredients of preCICE: communication, coupling schemes, and data mapping. To further increase the sustainability of preCICE, we will build on and extend the system test concept introduced in Section 6.3.

## Data Availability

Archived data at the time of publication is Test Setup of Turbine Blade Mapping Data ^56^. It consists of test setup and data to reproduce results in Section 3.2. This archive contains the following underlying data: Files array.sbatch and test.sbatch are job scripts used to execute the simulations on computing cluster systems. Folder meshes contains the mesh files which have coordinate and connectivity information of the geometries used in the simulations. Folder results contains CSV files having the results from which the plots in Section 3.2 are generated. File README.md contains steps to run the simulations and generate the results Data are available under the terms of the Creative Commons Attribution 4.0 International (CC BY 4.0)

## Software availability

Software and source code available from: https://github.com/precice Archived source code at time of publication is preCICE Distribution Version v2104.0 ^19^. Software in this project contain the following items and licenses:

- preCICE: LGPL-3.0 License
- Python bindings: LGPL-3.0 License
- Fortran module: LGPL-3.0 License
- Matlab bindings: LGPL-3.0 License
- OpenFOAM adapter: GPL-3.0 License
- deal.II adapter: LGPL-3.0 License
- FEniCS adapter: LGPL-3.0 License
- CalculiX adapter: GPL-3.0 License
- SU2 adapter: LGPL-3.0 License
- code_aster adapter: GPL-2.0 License
- Tutorials: LGPL-3.0 License
- vm: MIT License
- precice.github.io: CC BY 4.0 License
