# Supplementary material for: preCICE v2: A sustainable and user-friendly coupling library
Source: Open Res Eur. 2022 Sep 30;2:51. Originally published 2022 Apr 29. [Version 2] doi: 10.12688/openreseurope.14445.2 (PMC10446068; doi:10.12688/openreseurope.14445.2)
Supplement: Peer review history

# Peer review: Reviewer response for version 1

**DOI:** 10.21956/openreseurope.15594.r29132

**Published:** 2022-06-28

**Authors:** Garth Wells

**Affiliations:** 1 Department of Engineering, University of Cambridge, Cambridge, UK

This paper describes developments in the widely used, open-source preCISE library for coupling simulation codes. It also details development practices and community building. It is a worthwhile and valuable contribution.

 My main suggestion is to shorten the paper to better highlight the important and novel aspects. The paper is long, and I feel that the more important points are lost amongst standard and less important material. For example, the section on a virtual machine on page 16 could be reduced to one or two sentences. The description of each adapter in Section 4 is long and with detail, but without enough detail to describe each adaptor fully (which would not be reasonably possible). The descriptions could be considerably shorter whilst still conveying the main messages. Also, with careful editing the paper could be shortened considerably, even without removing any detail.

 The discussion on interpolation methods in Section 3.2 starts with the presumption of data defined at vertices. It is not clear from the text how it works with methods that do not use vertex degrees-of-freedom or how it works for methods where degrees-of-freedom are not point evaluations, or how higher-order methods are handled. It would be helpful if any limitation could be clearly stated, or if more general cases are supported then that could be explained. It would also be helpful in this section if conservation could be considered more formally.

 There are unsupported assertions in numerous places that could be removed without comprising the main messages of the paper, e.g. on p. 3 ". . . in the most important languages used in scientific computing" could be rephrased as ". . . in widely used languages for scientific computing". In other places judgements on the value/importance/significance of contributions are made rather than plain statements of what has been done. The text should be carefully reviewed for unsupported assertions and value judgements.

 Claims involving relative terms should be carefully reviewed, e.g. the last paragraph of Section 3.2 says a method "should only be used for small mesh sizes". Does this mean a mesh with few cells or a mesh with a large number of small cells? Given that 'small' is a relative term, what does the conclusions really mean? 

 It should be checked that all symbols are defined following their introduction in an equation.

Is the rationale for developing the new software tool clearly explained?

Yes

Is the description of the software tool technically sound?

Yes

Are sufficient details of the code, methods and analysis (if applicable) provided to allow replication of the software development and its use by others?

Yes

Is sufficient information provided to allow interpretation of the expected output datasets and any results generated using the tool?

Yes

Reviewer Expertise:

Scientific computing, mathematical software

I confirm that I have read this submission and believe that I have an appropriate level of expertise to confirm that it is of an acceptable scientific standard.

# Response

**Published:** 2022-09-14

**Authors:** Benjamin Uekermann

**Affiliations:** University of Stuttgart, Germany

Thank you for the thorough review and the constructive feedback. We see the point that the paper seems too long. We tried to address this issue in two ways: Firstly, we did indeed try to shorten the paper where reasonable. Section 4 had indeed too many details that users could also look up in the software documentation. Secondly, we now try to better guide readers in the introduction on what to read when. Concerning data mapping: preCICE does indeed expect data to be located at vertices. However, as vertices, a user could also define cell centers, for instance. Multiple meshes per participant are supported. preCICE does however not yet offer a good way how to deal with higher-order methods. We are tackling this issue in current work by allowing users to shift the data mapping from preCICE to the adapters. We added comments in the introduction of Section 3.2 to make these limitations and also the type of currently supported conservation clearer. We carefully reviewed the manuscript for unsupported assertions and value judgments and removed them.

# Peer review: Reviewer response for version 1

**DOI:** 10.21956/openreseurope.15594.r29131

**Published:** 2022-06-24

**Authors:** Axelle Viré

**Affiliations:** 1 Faculty of Aerospace Engineering, Delft University of Technology, Delft, The Netherlands

The paper is very interesting, relevant, and well written. I suggest minor revisions.

- Comment on the feasibility to couple in space of different dimensions, e.g., mapping from volume to surface or surface to line, and vice-versa. This is relevant for a range of applications but not covered at all as far as I can see.
- Is it possible to couple GPU tools or is it restricted to CPU-based models?
- Section 3.2.2 - Can you comment on the consistent or conservative character of this method?
- The test methodology is clearly described. But what about the review process, i.e. is there a review process when users or community members want to push new implementations in preCICE (e.g. for consistency of code syntax)?
- Checkpoints and restarts are not described it seems. I assume this is all possible and works well?
- Are the post-processing scripts shared as well for the tutorial cases?

**Minor editing comments:**

- References to bibliography are not always consistent in style, e.g., page 5 "introduced in 6".
- Page 3: 10,000s --> write in words so that the "s" is not mistaken for a unit.
- Ensure that all variables in equations are defined, even if straightforward, e.g. very first eq on page 5, distinction of superscripts and subscripts on page 7, etc.
- Page 7: IQN-IMVJ: time step, an idea --> add "following" before an idea.

Is the rationale for developing the new software tool clearly explained?

Yes

Is the description of the software tool technically sound?

Yes

Are sufficient details of the code, methods and analysis (if applicable) provided to allow replication of the software development and its use by others?

Yes

Is sufficient information provided to allow interpretation of the expected output datasets and any results generated using the tool?

Yes

Reviewer Expertise:

Fluid-structure interactions

I confirm that I have read this submission and believe that I have an appropriate level of expertise to confirm that it is of an acceptable scientific standard.

# Response

**Published:** 2022-09-14

**Authors:** Benjamin Uekermann

**Affiliations:** University of Stuttgart, Germany

Thank you for the thorough review and the positive words.

- Comment on the feasibility to couple in space of different dimensions (...)

We refer to such mixed-dimensional problems as geometric multi-scale coupling (following the definition of Quateroni *et al.* doi: 10.1016/j.cma.2016.01.007). Such problems can already now be realized by implementing the data mapping between different dimensions within the adapter (see for example Schmidt *et al*. doi: 10.1007/s10596-021-10120-8). We are currently working on dedicated geometric multi-scale data mapping methods within preCICE as mentioned in the conclusions. We extended this part of the conclusions.

- Is it possible to couple GPU tools or is it restricted to CPU-based models?

Yes, hybrid GPU-CPU coupling is possible. A few users have done this already. We added an explanation and mention some examples in the beginning of Section 3.

- Section 3.2.2 - Can you comment on the consistent or conservative character of this method?

We present and compare all data mapping methods in their consistent variant. By transposing the corresponding system matrix a consistent method turns conservative. We added additional explanation in the introduction part of Section 3.2, trying to point out more clearly what we mean with consistency and conservation in our context and what the restrictions are. In addition, we comment on the realization of consistency and conservation in RBF data mapping methods at the end of Section 3.2.2.

- The test methodology is clearly described. But what about the review process, i.e. is there a review process when users or community members want to push new implementations in preCICE (e.g. for consistency of code syntax)?

Every contribution is reviewed for functionality (does the code run and do the expected thing?), coding practices (is the code clear and reasonable?), consistency (does the implementation follow similar solution patterns?), and usability (is the interface intuitive and sufficiently documented?). Pull request templates in each project remind the authors and the reviewers of common issues to check. While these templates contain checklists, these are not extensive, to reduce the overhead in smaller contributions. We added a note in Section 7.

- Checkpoints and restarts are not described it seems. I assume this is all possible and works well?

To restart a coupled simulation, typically all coupled codes need to read checkpoints and restart. preCICE directly supports this behavior without any modifications. preCICE itself, however, does (currently) not write checkpoints for restarting. This means, for instance, that the quasi-Newton system starts from scratch again and that data mappings need to be recomputed. For most applications, this behavior is sufficient. If ever necessary, adding such a checkpoint-restart mechanism to preCICE itself should, however, be possible.

- Are the post-processing scripts shared as well for the tutorial cases?

Yes, they are. We write at the beginning of Section 4: "Such a tutorial consists of all the required instructions and configuration files necessary to run the coupled simulation, as well as convenience scripts to run, visualize, and cleanup each case."
